# Study of a precise treatment protocol for patients with consciousness disorders based on the brain network analysis of functional magnetic resonance imaging

**DOI:** 10.3389/fnins.2024.1443478

**Published:** 2024-09-16

**Authors:** Pingzhi Wang, Jie Xiang, Yan Niu, Jing Wei, Caiqin Lan, Xiangping Li, Liying Xu, Yajie Yin, Hongxiong Wang, Tao Zhang, Lei Yang, Hao Xing, Shasha Fan, Qing Niu, Huicong Kang, Ying Liang

**Affiliations:** ^1^Third Hospital of Shanxi Medical University, Shanxi Bethune Hospital, Tongji Shanxi Hospital, Shanxi Academy of Medical Sciences, Taiyuan, China; ^2^College of Computer Science and Technology (College of Data Science), Taiyuan University of Technology, Taiyuan, China; ^3^Tongji Medical College, Tongji Hospital, Huazhong University of Science and Technology, Wuhan, China

**Keywords:** disorders of consciousness, brain network, functional magnetic resonance imaging, olfactory cortex, repeated transcranial magnetic stimulation, occipital lobe

## Abstract

**Objective:**

How to conduct objective and accurate individualized assessments of patients with disorders of consciousness (DOC) and carry out precision rehabilitation treatment technology is a major rehabilitation problem that needs to be solved urgently.

**Methods:**

In this study, a multi-layer brain network was constructed based on functional magnetic resonance imaging (fMRI) to analyze the structural and functional brain networks of patients with DOC at different levels and to find regulatory targets (imaging markers) with recovery potential for DOC. Then repeated transcranial magnetic stimulation (rTMS) was performed in DOC patients to clinically validate.

**Results:**

The brain network connectivity of DOC patients with different consciousness states is different, and the most obvious brain regions appeared in the olfactory cortex and precuneus. rTMS stimulation could effectively improve the consciousness level of DOC patients and stimulate the occipital lobe (specific regions found in this study) and the dorsolateral prefrontal cortex (DLPFC), and both parts had a good consciousness recovery effect.

**Conclusion:**

In clinical work, personalized stimulation regimen treatment combined with the brain network characteristics of DOC patients can improve the treatment effect.

## 1 Introduction

Consciousness is a multi-layered concept primarily consisting of wakefulness and awareness. DOC refers to a pathological state characterized by dissociation between wakefulness and awareness. Based on the level of consciousness, DOC can be further categorized into Vegetative State/Unresponsive Wakefulness Syndrome (VS/UWS), Minimally Conscious State (MCS), and Emerging from Minimally Conscious State (EMCS). Patients with DOC exhibit varying levels of awareness and responsiveness. While Coma patients cannot be awakened by external stimuli, there is no normal sleep awakening cycle rhythm; VS/UWS patients retain sleep-wake cycles and basic reflex behaviors but lack awareness of the external environment and self; MCS patients display non-reflexive autonomous behaviors with slight but inconsistent awareness of the surroundings or self. EMCS patients can use objects and engage in functional communication. On average, lifetime care costs for DOC patients range from $600,000 to $1,875,000, with an average of around $1,000,000 per person. This not only imposes significant financial burdens on patients' families but also represents a substantial burden on society and the nation (Consensus Conference, 1999). In clinical practice, the diagnosis of the level of consciousness in DOC patients mainly relies on behavioral assessment scales. Commonly used scales include the Glasgow Coma Scale (GCS) and the Coma Recovery Scale-Revised (CRS-R) (Teasdale and Jennett, 1976). Although behavioral assessment scales are cost-effective, easy to operate, and widely used in clinical practice, the assessment results are easily influenced by the experience and subjectivity of the assessing physician. Additionally, motor and speech impairments are very common in patients with DOC, and reliance on behavioral responses for assessment may underestimate the true level of consciousness in DOC patients. According to statistics, diagnoses of consciousness levels based solely on clinical observation have an error rate as high as 40% (Wannez et al., 2017). Therefore, there is an urgent need to find a new medical approach that can accurately diagnose and effectively treat these disorders, which will not only help in the recovery of patients' consciousness and functionality but also lead to further savings in social, medical, and economic resources. The European guidelines on consciousness disorders released in 2020 recommend that the most accurate diagnostic method for clinical consciousness disorders is a combination of behavioral assessment, neurophysiological examination, and neuroimaging evaluation (Kondziella et al., 2020).

The human brain contains a vast number of neurons that are interconnected, forming an extremely complex brain network structure. With the continuous development of computer science and artificial intelligence technology, people began to study the connections and interactions between different brain areas in the brain and use noninvasive means to measure the changes in blood oxygenation levels during brain activity to indirectly reflect brain neuronal activity. This avoids many situations that are not clinically observed. In 2006, Owen and Coleman (2006) first reported that during the motor imagery and spatial localization tasks, a patient clinically diagnosed with VS/UWS coincided with healthy controls, proving that the patient had perceived in both the self and external environment, and a large cohort study showed that this phenomenon was not accidental.

The construction of functional brain networks based on neuroimaging data has become a hot topic in the field of neuroscience. Previous healthy-people resting-state fMRI research has confirmed the existence of large-scale functional networks. Research shows that consciousness in multiple brain network structures, including the frontal-parietal and default mode networks (DMN), plays a key role in the process of consciousness in a specific neural network, as well as in self-consciousness, introspection, psychological simulation, and other cognitive functions. DMN is active in the brain and decreases activity during cognitive task execution. The current fMRI study shows widespread abnormalities in brain regions and functional networks (Bodien et al., 2017), mainly in the DMN in the posterior cingulate cortex (PCC) and medial prefrontal cortex (mPFC), as well as other external networks involved in processing sensory stimuli and performing higher-order cognitive tasks (Qin et al., 2015; Wu et al., 2015). However, in clinical work, it is common for patients with similar conditions but far from clinical behavioral manifestations. The rapid advancement of brain networks holds promise for identifying neuroimaging biomarkers that can effectively differentiate between different levels of consciousness in DOC patients and provide new neurophysiological clues for the occurrence and development of consciousness disorders.

In 2023, the first affiliated hospital of Nanjing Medical University (Jiangsu, China) conducted a systematic evaluation (Liu et al., 2023) showing that transcranial direct current stimulation (tDCS) can effectively improve the level of consciousness of patients with chronic consciousness disorder. The etiology of consciousness disorder, consciousness disorder course and consciousness level are important factors affecting the efficacy of non-invasive brain stimulation technology, which further provides an important reference for the clinical application of non-invasive brain stimulation technology. However, at present, there is less clinical evidence of efficacy, type selection, stimulation target, stimulation frequency, and intensity optimization (Pistarini and Maggioni, 2018). Given the large number of patients with DOC and the huge treatment demand, further research is needed to find more accurate treatment targets and develop more objective and effective treatment plans.

Currently, fMRI, combined with clinical information and other features, uses the brain connectivity network algorithm to predict therapeutic targets and conduct clinical validation. This model has been developed and applied and is expected to realize the accurate diagnosis and prognostic evaluation of DOC and provide an effective basis for the formulation of the next treatment plan and the precise positioning of neuroregulation.

In conclusion, by combining fMRI with graph theory analysis, this paper explored the brain network topological organization characteristics and possible pathogenesis of DOC patients from the whole-brain functional network level to screen out objective markers and determine the best stimulating brain areas. Through the implementation of rTMS treatment, observing the changes in its before and after efficacy, and the in-depth analysis of the structural and functional characteristics of brain networks, we can deepen our understanding of the mechanism of rTMS treatment awareness disorders and provide important technical support and method guidance for the precise treatment of rTMS in DOC patients in clinical practice.

## 2 Materials and methods

### 2.1 Participants

#### 2.1.1 Brain network research

A total of 47 patients with DOC who were admitted to the Rehabilitation Medicine Department of Bethune Hospital in Shanxi Province from November 2021 to March 2023 were included in the study and underwent MRI scanning. Patient inclusion criteria: meet the diagnostic criteria for disorder of consciousness; use the CRS-R scale to evaluate and meet the diagnostic standards of VS and MCS; no contraindications to MRI scanning; no history of mental illness, alcoholism, or drug abuse; no severe brain atrophy or deformation; and legal guardian of the patient those who signed the informed consent form and were willing to receive follow-up visits.

Before the MRI scan, two experienced rehabilitation medicine doctors who had undergone standardized training used the GCS and the CRS-R scales to assess the level of consciousness of patients with DOC for five consecutive days. The GCS is a commonly used scale in clinical practice to assess the level of consciousness in patients, consisting of three sub-items: eye response, verbal response, and motor response. The CRS-R is currently considered the “gold standard” for assessing the level of consciousness in patients with DOC and is widely used in clinical and scientific research, mainly comprising six sub-items: auditory, visual, motor, oromotor, communication, and arousal functions, ranging from reflexive activity to cognitively mediated behaviors. Based on the assessment results, DOC patients can be classified into three categories: VS, MCS, and EMCS (Specific criteria are provided in Appendix Figure A1). Patients who required anesthesia or sedation during the scanning process and had a head movement >2 mm on imaging were excluded. Ultimately, data from 23 DOC patients were retained. Based on behavioral assessments, 11 patients were diagnosed with VS/UWS, and 12 patients were diagnosed with MCS. Additionally, we recruited 28 age- and gender-matched healthy volunteers to serve as a healthy control group (HC). Inclusion criteria: there are no contraindications to MRI scanning; no history of neurological or mental illness, alcoholism, or drug abuse; and no systemic disease.

#### 2.1.2 rTMS research

DOC patients hospitalized in the Rehabilitation Medicine Department of Shanxi Bethune Hospital from June 2023 to February 2024 were selected as the study subjects. The patient inclusion criteria were as follows: 1. 18–70 years old, with acquired brain injury < 1 year; 2. no previous neuropsychiatric-related diseases; 3. no sedative drugs that may interfere with brain stimulation, such as Na+ or Ca+ channel blockers or N-methyl-D-aspartic acid (NMDA) receptor antagonists; 4. stable vital signs; 5. the family members voluntarily participated in the study and provided signed informed consent. The exclusion criteria were as follows: 1. patients participating in other non-invasive or invasive neuromodulation tests; 2. uncontrolled seizures, that is, seizures within 4 weeks before enrollment; 3. contraindications to rTMS, such as skull metal implantation, pacemaker, stimulation site craniotomy, etc.

This study was approved by the Ethics Committee of Shanxi Bethune Hospital (No. SBQKL-2021-021) and obtained written informed consent from each healthy volunteer and the legal guardian of the patient following the Helsinki Declaration.

### 2.2 Data acquisition

All MRI data were acquired using SIEMENS MAGNETOM Skyra equipment equipped with standard transmit-receive head coils. All participants were positioned in a supine position on the MRI examination table. The HC was instructed to maintain visual and auditory isolation and to relax their whole body. To minimize motion artifacts, sponge strips were placed on both sides of the heads of all participants for head fixation. Resting-state fMRI data was acquired using a single-shot multi-slice gradient-echo echo-planar imaging (GE-EPI) sequence. The sequence parameters are as follows: repetition time (TR) = 3,000 ms, echo time (TE) = 30 ms, flip angle (FA) = 90°, field of view (FOV) = 192 × 192 mm^2^, data matrix = 94 × 94, slice thickness = 1.5 mm, slice gap = 0.4 mm, 72 axial contiguous slices, acquired 90 volumes in ~5 min. High-resolution structural brain images were obtained using a T1-weighted 3D fast spoiled gradient-recalled (T1-3D-MPAGE) sequence with the following parameters: TR = 2,000 ms, TE = 2.98 ms, FOV = 256 × 256 mm^2^, data matrix = 256 × 256, FA = 9, voxel size = 1 × 1 × 1 mm^3^, covering 192 sagittal slices across the whole brain.

### 2.3 Data processing

This study utilized the Data Processing Assistant for Resting-State fMRI (DPARSF) module from Data Processing and Analysis for Brain Imaging (DPABI) to preprocess fMRI data. DPARSF preprocesses images based on the algorithms from Statistical Parametric Mapping (SPM) (http://www.fil.ion.ucl.ac.uk/spm). The process includes removing the first 10 time points to achieve T1 equilibrium. Perform slice timing and realignment on the remaining images. The T1-weighted images were coregistered with functional images and divided into gray matter (GM), white matter (WM), and cerebral spinal fluid (CSF) tissue maps by Diffeomorphic Anatomical Registration Through Exponentiated Ligalgebra (DARTEL). Finally, these processed images were spatially normalized to the Anatomical Automatic Labeling (AAL) space provided by the Montreal Neurological Institute (MNI). The voxels were resampled as 3 mm × 3 mm × 3 mm, and a Gaussian smoothing kernel with a full width at half maxima (FWHM) of 6 mm was used for smoothing to enhance the signal-to-noise ratio. The above image preprocessing steps prepare the data to meet the requirements for subsequent statistical analysis.

### 2.4 Functional brain network analysis

Graph theory is a powerful tool for analyzing brain connectivity, which can characterize the overall or local behavior of brain networks. This study utilizes graph theory metrics to analyze the structural and functional connectivity of brain networks. Imaging preprocessing is carried out using DPABI and SPM, while network construction and analysis are conducted using the Graph Theoretical Network Analysis (GRETNA) toolbox. This study utilized the AAL brain parcellation template established by the MNI to construct brain networks. Each participant's brain was segmented into 90 brain regions, with 45 regions on each hemisphere (see Appendix Figures B1–3). Each brain region serves as a network node, and the average time series of all voxels in each brain region was calculated based on preprocessed image data. The functional brain network can be divided into two categories: global analysis and local analysis. The global analysis metrics include small-world and network efficiency; the local analysis metrics include betweenness centrality, degree centrality, Nodal clustering coefficient, and Nodal efficiency.

### 2.5 Randomization allocation scheme for rTMS

With the help of the random genus function of the statistical software SPSS 26.0, the computer automatic randomization grouping was realized to ensure the impartiality and objectivity of the grouping. Patients were randomly assigned to the two groups in a 1:1 ratio, and this randomization process was carried out by the professional staff of the hospital. The blind code group is safely placed in a closed, opaque envelope to ensure the effective implementation of the blind method.

The rTMS treatment process was independently completed by rehabilitation therapists with professional qualifications from the Rehabilitation Medicine Department of Shanxi Bethune Hospital, who focused on giving accurate rTMS stimulation to patients to ensure the standardization and safety of the treatment process. These therapists will not be aware of or participate in any efficacy assessment work throughout the trial to ensure the objectivity and impartiality of the assessment results.

However, the efficacy evaluation of patients was conducted by two qualified attending physicians, who completed the evaluation of the main evaluation indicators before and after treatment. Such arrangements are designed to ensure the independence and professionalism of the assessment process and yield more accurate and reliable efficacy assessment results.

### 2.6 The rTMS parameter scheme

The transcranial magnetic stimulation (TMS) device used in this study was a MagTD stimulator (Wuhan Yide Company, China), equipped with an outer diameter of 87 mm and an 8-shaped coil, which can produce a bipolar pulse width of 0.1 ms.

The resting motor threshold (RMT) was measured for each subject during the first treatment, and the RMT determined the magnetic stimulation intensity of the patient. The most commonly used in clinical and scientific research was 80% to 120% motor threshold (MT).

**Figure 1:**
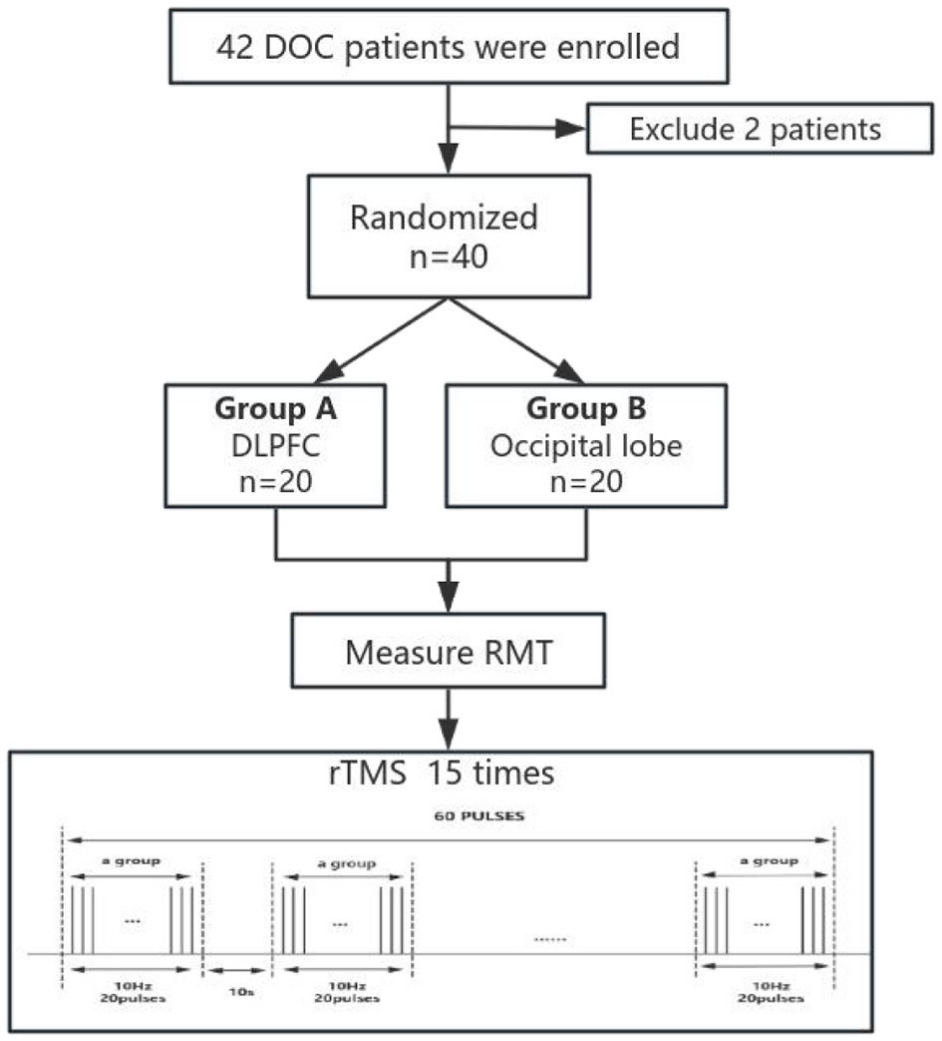
A schematic diagram of the rTMS research.

A single rTMS treatment contained 60 treatment groups with 20 pulses at 10 Hz. The interval between the groups was 10 s, and 1,200 pulses were output throughout the treatment process, accounting for a total duration of 12 min. The intensity of the rTMS during treatment was set at 100% of the individual MT to ensure the maximization of treatment effects. For the grouping of stimulated subjects, the left dorsolateral prefrontal region, or occipital lobe, was selected for precise stimulation (Figure 1).

### 2.7 Basic treatment and routine rehabilitation

All the subjects participating in this study received routine drug treatment, examination and evaluation, professional nursing, and a series of rehabilitation treatment programs at the Rehabilitation Medicine Department of Shanxi Bethune Hospital. Among them, rehabilitation treatment programs include electric standing bed training, hyperbaric oxygen therapy, traditional rehabilitation treatment, respiratory function rehabilitation, limb active and passive movement, and others.

### 2.8 Statistical analysis

This study utilized SPSS 26.0 software for statistical analysis.

Brain network analysis: The demographic data were expressed as mean ± standard deviation, and the age difference between the DOC and HC groups was compared by an independent sample *t*-test. The chi-square test was used to compare the gender differences between the two groups. An independent sample *t*-test was used to compare the brain function data within the two groups (such as HC and VS), and a one-way analysis of variance (ANOVA) was used to compare the brain function data within the three groups (VS, MCS, and HC).

rTMS analysis: data meeting the normal distribution were represented by mean ± standard deviation; data that did not meet the normal distribution were represented by median (interquartile spacing), Mann–Whitney U test, and Wilcoxon signed-rank test for intra-group comparison. In this study, *P* < 0.05 was considered statistically significant.

## 3 Results

### 3.1 Brain network analysis

#### 3.1.1 Demographic and clinical statistics

**Figure 2:**
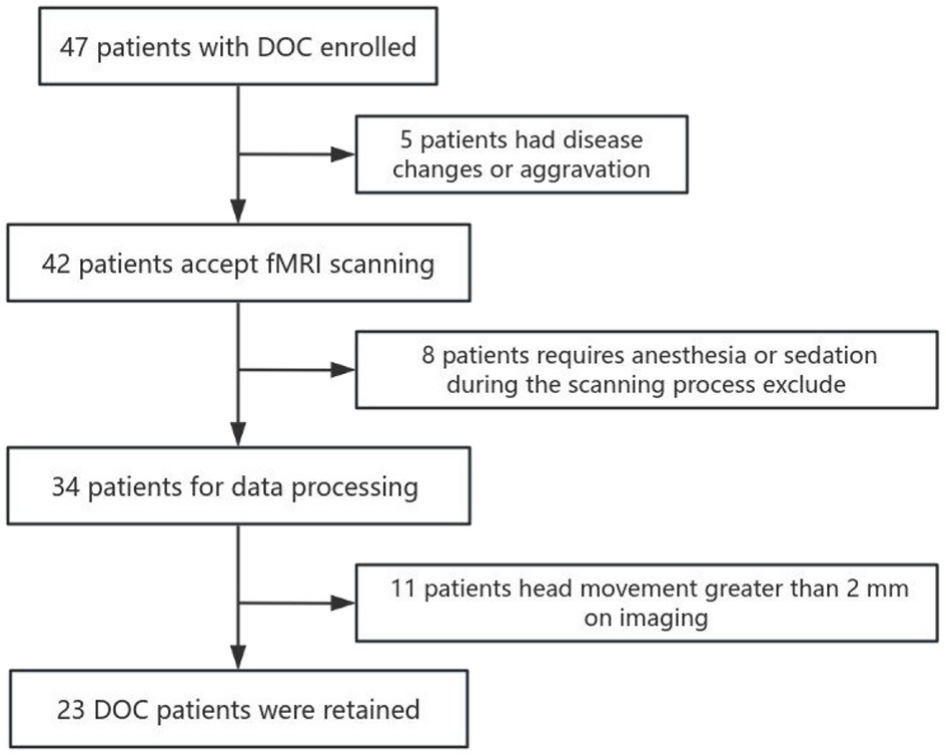
Flow chart of the DOC patients enrolled in the brain network research.

This study included a total of 47 DOC patients who were admitted to Shanxi Bethune Hospital from November 2021 to March 2023 and underwent MRI scans. Among them, seven patients received sedative drugs during the scanning process, 11 patients had blurry, deformed, or severely hydrocephalic high-resolution T1 structural images and 6 patients had head motion images >2 mm, all of which were excluded. Therefore, a total of 23 patients were included in the analysis (Figure 2). In addition, we included 28 healthy subjects matched for age and gender as a control group. Among the 23 DOC patients included in the analysis, 11 were male and 12 were female, with an average age of 55.96 ± 9.167 years. This group comprised 8 patients with traumatic brain injury, 11 patients with cerebrovascular accidents, two patients with ischemic-hypoxic brain disease, and two other patients with demyelinating diseases and postoperative meningioma (Table 1). In the included 28 healthy controls, 14 were male and 14 were female, with an average age of 56.35 ± 10.530 years. Statistical analysis revealed no significant differences between the DOC group and the HC group in terms of age (*F* = 1.346, *P* = 0.252) and gender (X^2^ = 0.024, *P* = 0.877) (Table 2).

**Table 1:** Details the clinical characteristics of each DOC patient, including age, sex, etiology, disease duration, CRS-R score, and clinical diagnosis.

**ID**	**Sex**	**Age**	**Etiology**	**Lesion information**	**Days since onset**	**CRS-R scores; Aud/Vis/Mot/ Oro/Com/Aro/T**	**GCS E/V/M**	**Diagnosis**
1	Male	42	TBI	Bilateral frontal and temporal lobes	27	3/1/2/0/0/2/8	4/T/4	VS
2	Male	62	CVA	Basal ganglia	28	0/0/0/0/0/2/2	4/T/1	VS
3	Male	69	CVA	Basal ganglia	80	1/2/2/1/0/2/8	4/T/4	VS
4	Female	45	CVA	Left frontotemporal parietal lobe	47	2/3/1/1/0/2/9	4/T/3	VS
5	Female	70	CVA	Basal ganglia	35	2/3/5/0/0/2/12	4/T/4	VS
6	Female	69	CVA	Bilateral parietal lobe	31	1/0/2/0/0/2/5	4/1/4	VS
7	Female	50	TBI	Right frontotemporal roof	25	0/0/2/0/0/1/3	2/T/4	VS
8	Female	53	Other	Meningioma resection	75	0/1/2/0/0/2/5	4/T/4	VS
9	Male	36	TBI	Left frontal, temporal, parietal, and occipital	29	0/0/2/0/0/0/2	1/T/4	VS
10	Male	46	TBI	Right frontal, parietal, and temporal	65	1/0/5/0/0/2/8	4/T/4	VS
11	Male	66	TBI	Basal ganglia	50	0/0/2/0/0/1/3	2/T/4	VS
12	Male	71	TBI	Subarachnoid hemorrhage	20	0/0/5/0/0/2/7	4/T/4	MCS
13	Female	61	CVA	Left frontal lobe, cerebellar hemisphere, pons	59	0/0/1/0/0/2/3	4/T/2	MCS
14	Male	65	HIE	Bilateral frontal, temporal, parietal, and occipital	25	0/0/2/0/0/2/4	4/T/4	MCS
15	Female	65	CVA	Right frontal, temporal, parietal, and occipital	52	1/0/2/1/0/2/6	4/1/4	MCS
16	Male	42	TBI	Demyelinating disease	14	4/5/5/0/1/3/19	4/T/6	MCS
17	Male	66	CVA	Basal ganglia	55	1/2/1/0/0/2/6	4/T/2	MCS
18	Female	61	CVA	Basal ganglia	130	0/0/2/0/0/1/3	2/T/4	MCS
19	Female	52	TBI	Demyelinating disease	21	0/0/2/0/0/2/4	4/T/4	MCS
20	Female	54	HIE	Brainstem	54	1/2/5/1/0/2/11	4/T/4	MCS
21	Female	54	Other	Demyelinating disease	82	2/3/2/0/0/2/9	4/T/4	MCS
22	Female	53	CVA	Brainstem	59	1/3/2/0/0/2/8	4/T/3	MCS
23	Male	44	CVA	Brainstem	128	3/4/5/1/0/2/15	4/1/6	MCS

TBI, Traumatic Brain Injury; CVA, Cerebral Vascular Accident; HIE, Hypoxic Ischemic Encephalopathy; CRS-R, Coma Recovery Scale Revised; Aud, auditory; Vis, visual; Mot, motor; Oro, oromotor; Aro, arousal; T, total CRS-R score; GCS, Glasgow coma scale; E, Eye opening; V, Verbal response; M, Motor response; VS, Vegetative state; MCS, Minimally conscious state.

**Table 2:** Comparison of demographic characteristics between the DOC group and HC group duration, CRS-R score, and clinical diagnosis.

**Characteristics**	**DOC**	**HC**	***P*-value**
Age	55.96 ± 9.167	56.35 ± 10.530	0.252^a^
Sex (M/F)	11/12	14/14	0.877^b^
Atiology (TBI/CVA)	8/11		
Diagnosis (VS/MCS)	11/12		

M, male; F, Female; TBI, Traumatic Brain Injury; CVA, Cerebral Vascular Accident; VS, Vegetative state; MCS, Minimally conscious state; ^a^two-sample t-test; ^b^χ^2^ test.

#### 3.1.2 Global brain network

**Figure 3:**
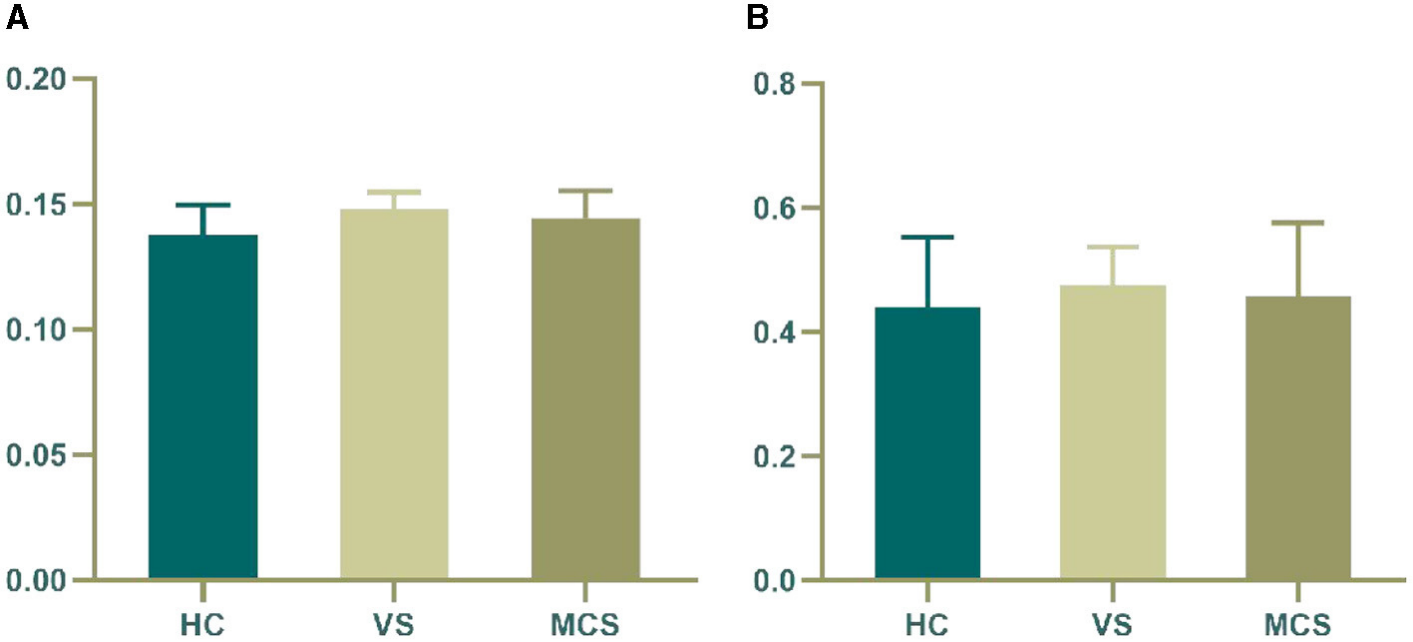
Differences in the global brain network. **(A)** network efficiency among the three groups; **(B)** small word among the three groups. The vertical axis indicates the functional connection strength.

In brain network analysis, network efficiency can be used to evaluate the efficiency of information transmission and interaction between brain regions, helping to understand the information processing mechanisms of the brain and the operational status of brain functions. The higher the network efficiency, the less energy is consumed in the interaction between nodes, making information transmission on the brain network more effective. A small word is an important concept to describe the structural characteristics of brain networks. It represents the dense connections formed by the highly aggregated nodes in the local scope of brain networks, which can efficiently transmit information in the global scope. This property has an important impact on the information communication and collaborative processing between brain regions, which helps to improve the operation efficiency and information processing ability of brain networks. *T*-tests and ANOVA revealed no significant differences in small word and network efficiency between the VS, MCS, and HC groups, both within and between groups (as shown in Figure 3, Table 3).

**Table 3:** Statistics of the global brain network.

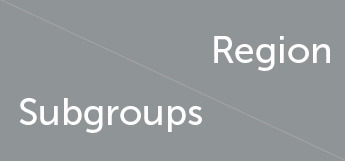	**Network efficiency**	**Small word**
HC	0.14 ± 0.01	0.44 ± 0.11
VS	0.15 ± 0.01	0.48 ± 0.06
MCS	0.14 ± 0.01	0.46 ± 0.12
F	2.129	0.261
*P*	0.138	0.772

#### 3.1.3 Local brain network

##### 3.1.3.1 Nodal betweenness centrality

**Figure 4:**
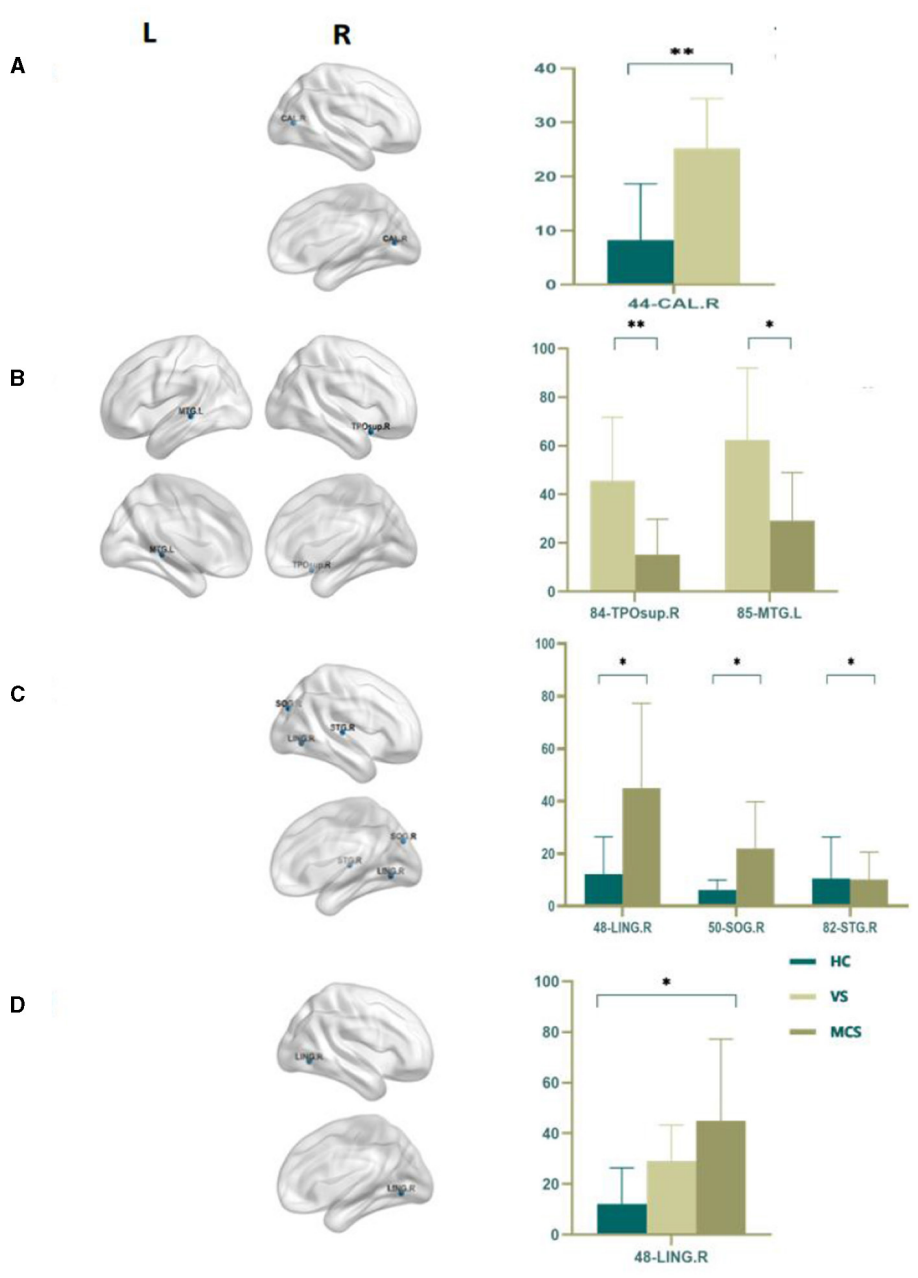
Differences in the Nodal betweenness centrality. **(A)** Comparison between HC and VS; **(B)** Comparison between VS and MCS; **(C)** Comparison between HC and MCS; **(D)** Comparison between three groups; The 3D brain model on the left presents the localization of brain regions featuring significant differences. The vertical axis indicates the functional connection strength; *P < 0.05; **P < 0.005.

Nodal betweenness centrality is a key metric for assessing the importance of nodes in brain networks. The higher the betweenness centrality, the greater the control role of this node in the network, which has an important impact on the fluency of information transmission. By using an independent sample *t*-test, we found that compared with the HC group, the VS group had higher Nodal betweenness centrality in the right calcarine fissure (44-CAL.R) (*P* = 0.003). In the MCS group, the brain areas with increased Nodal betweenness centrality are the right lingual gyrus (48-LING.R) (*P* = 0.006) and the right superior occipital gyrus (50-SOG.R) (*P* = 0.011), while the brain area with decreased Nodal betweenness centrality is the right superior temporal gyrus (82-STG.R) (*P* = 0.010); In comparison between the VS group and the MCS group, the brain regions with increased Nodal betweenness centrality are the right temporal pole: the superior temporal gyrus (84-TPOsup.R) (*P* = 0.004) and the left middle temporal gyrus (85-MTG.L) (*P* = 0.009). When comparing among the three groups, the brain region with differential Nodal betweenness centrality is the right lingual gyrus (48-LING.R) (*P* = 0.009) (as shown in Figure 4, Table 4).

**Table 4:** Statistics of Nodal betweenness centrality.

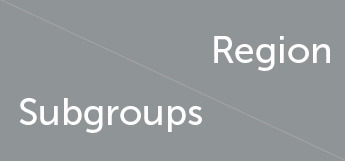	**44-CAL.R**	**48-LING.R**	**50-SOG.R**	**82-STG.R**	**84-TPOsup.R**	**85-MTG.L**
HC	8.265 ± 10.384	12.155 ± 14.249	6.155 ± 3.779	10.472 ± 15.867	-	-
VS	25.220 ± 9.199	29.100 ± 14.208	-	-	45.663 ± 26.071	62.371 ± 29.584
MCS	-	44.942 ± 32.295	21.984 ± 17.767	10.148 ± 10.448	15.206 ± 14.602	29.224 ± 19.731
*P*	0.003	0.006	0.011	0.01	0.004	0.009

Due to different statistical methods for inter-group and intra-group comparisons, the table presents the minimum p value between groups or within groups and the specific values are shown in the text.

##### 3.1.3.2 Nodal degree centrality

Nodal degree centrality is a measure of the importance of a node in a network, defining the centrality of a node in the network and reflecting its information processing and transmission capabilities within the local network.

In comparison to the HC group, the brain regions where patients in the VS group showed increased Nodal degree centrality are the right olfactory cortex (22-OLF.R) (*P* = 0.009), the right rectus gyrus (28-REC.R) (*P* = 0.008), the left hippocampus (37-HIP.L) (*P* = 0.005), the left parahippocampal gyrus (39-PHG.L) (*P* = 0.005), and the right caudate nucleus (72-CAU.R) (*P* = 0.031). The brain regions where Nodal degree centrality decreased are the left olfactory cortex (21-OLF.L) (*P* = 0.050), the right postcentral gyrus (58-PoCG.R) (*P* = 0.009), and the bilateral precuneus (67-PCUN.L, 68-PCUN.R) (*P* = 0.003). In MCS patients, brain regions with increased Nodal degree centrality include the right superior frontal gyrus (6-ORBsup.R) (*P* = 0.005), right middle frontal gyrus (8-MFG.R) (*P* = 0.004), right olfactory cortex (22-OLF.R) (*P* = 0.008), right rectal gyrus (28-REC.R) (*P* = 0.006), and left caudate nucleus (71-CAU.L) (*P* = 0.002); brain regions with decreased Nodal degree centrality include the left superior occipital gyrus (49-SOG.L) (*P* = 0.008); and left superior parietal gyrus (59-SPG.L) (*P* < 0.0001). In comparison between the VS/UWS group and the MCS group, brain regions with increased Nodal degree centrality include the right insula (30-INS.R) (*P* = 0.009), while brain regions with decreased Nodal degree centrality include the left precuneus (67-PCUN.L) (*P* = 0.003).

**Figure 5:**
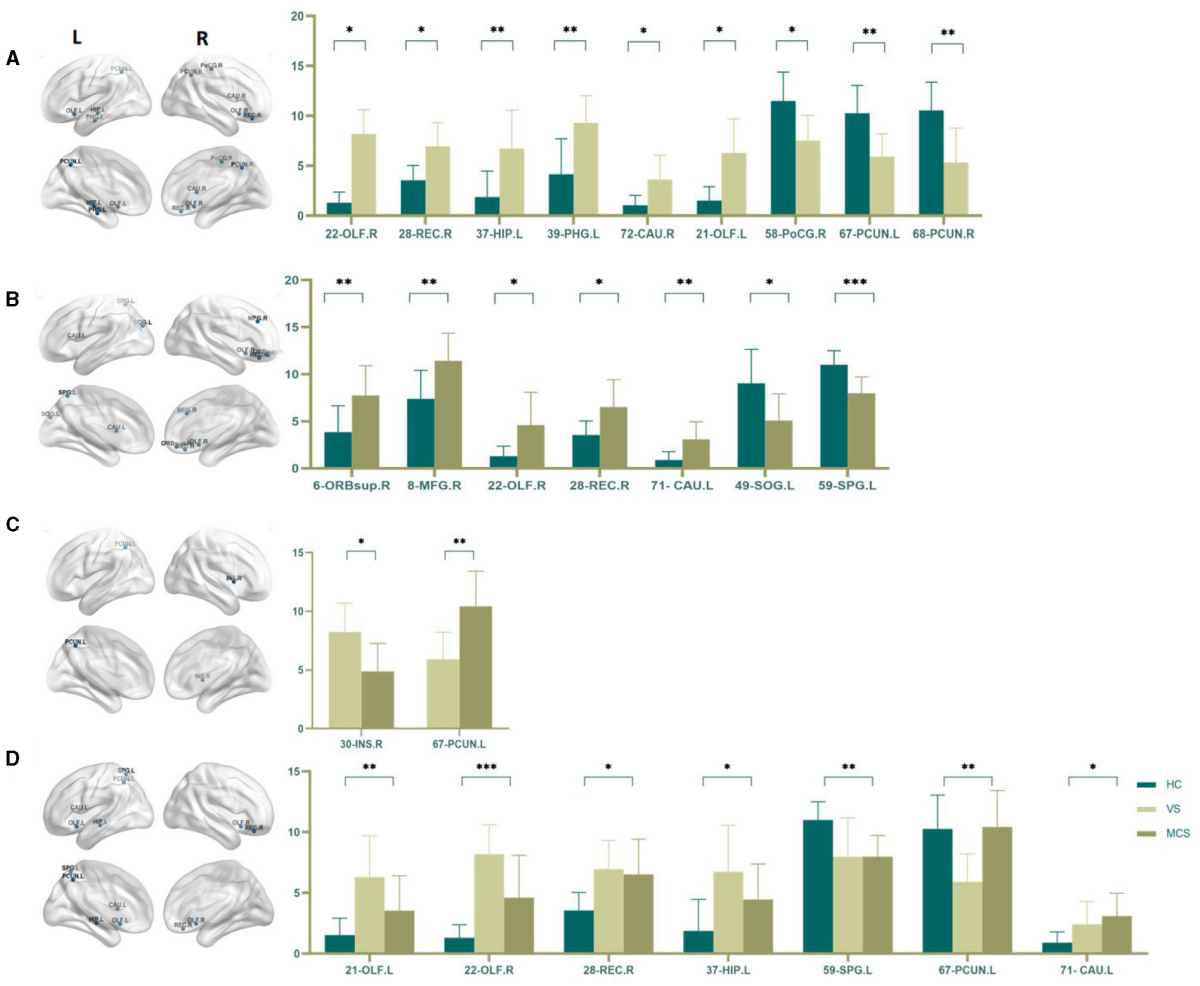
Differences in the Nodal degree centrality. **(A)** Comparison between HC and VS; **(B)** Comparison between HC and MCS; **(C)** Comparison between VS and MCS; **(D)** Comparison between three groups; The 3D brain model on the left presents the localization of brain regions featuring significant differences. The vertical axis indicates the functional connection strength; *P < 0.05; **P < 0.005; ***P < 0.001.

When comparing among the three groups, brain regions with differential Nodal degree centrality include the left olfactory cortex (21-OLF.L) (*P* = 0.003), right olfactory cortex (22-OLF.R) (*P* < 0.0001), right rectus gyrus (28-REC.R) (*P* = 0.006), left hippocampus (37-HIP.L) (*P* = 0.009), left superior parietal gyrus (59-SPG.L) (*P* = 0.003), left precuneus (67-PCUN.L) (*P* = 0.004), and left caudate nucleus (71-CAU.L) (*P* = 0.009) (as shown in Figure 5, Table 5).

**Table 5:** Statistics of Nodal degree centrality.

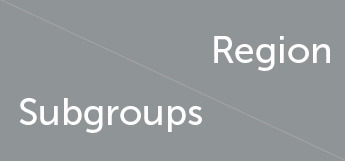	**6-ORBsup.R**	**8-MFG.R**	**21-OLF.L**	**22OLF.R**	**28 REC.R**	**30- INS.R**	**37- HIP.L**	**39- PHG.L**
HC	3.854 ± 2.805	7.389 ± 3.012	1.528 ± 1.378	1.304 ± 1.073	3.560 ± 1.483	-	1.873 ± 2.594	4.163 ± 3.540
VS	-	-	6.297 ± 3.410	8.193 ± 2.425	6.951 ± 2.368	8.237 ± 2.464	6.744 ± 3.840	9.316 ± 2.728
MCS	7.765 ± 3.144	11.449 ± 2.928	3.540 ± 2.863	4.606 ± 3.490	6.526 ± 2.906	4.875 ± 2.387	4.453 ± 2.933	-
*P*	0.005	0.004	0.003	< 0.0001	0.006	0.009	0.005	0.005
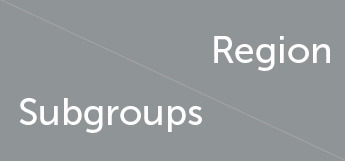	**49-SOG.L**	**58-PoCG.R**	**59-SPG.L**	**67- PCUN.L**	**68- PCUN.R**	**71- CAU.L**	**72- CAU.R**	
HC	9.045 ± 3.609	11.515 ± 2.874	11.010 ± 1.493	10.269 ± 2.782	10.550 ± 2.828	0.900 ± 0.891	1.051 ± 0.994	
VS	-	7.526 ± 2.554	7.991 ± 3.208	5.917 ± 2.296	5.337 ± 3.454	2.416 ± 1.866	3.629 ± 2.426	
MCS	5.098 ± 2.832	-	7.988 ± 1.738	10.435 ± 3.002	-	3.091 ± 1.872	-	
*P*	0.008	0.009	< 0.0001	0.003	0,003	0.002	0.031	

Due to different statistical methods for inter-group and intra-group comparisons, the table presents the minimum p value between groups or within groups and the specific values are shown in the text.

##### 3.1.3.3 Nodal clustering coefficient

The Nodal clustering coefficient is a local feature of the brain network, indicating the ratio of the actual number of edges connecting a node to its neighboring nodes to the maximum possible number of connecting edges. It is a measure of the clustering level of nodes in the network, used to assess the clustering degree and local information transmission efficiency of different brain regions in neural information transmission. In comparison to the HC group, the brain regions where the Nodal clustering coefficient increased in the VS/UWS group patients were the right orbital part of the inferior frontal gyrus (16-ORBinf.R) (*P* = 0.001), the right olfactory cortex (22-OLF.R) (*P* = 0.009), and the left hippocampus (37-HIP.L) (*P* = 0.005); while the brain regions where the Nodal clustering coefficient decreased included the right postcentral gyrus (36-PCG.R) (*P* = 0.005), the right cuneus (46-CUN.R) (*P* = 0.004), the left lingual gyrus (47-LING.L) (*P* = 0.004), the right lingual gyrus (48-LING.R) (*P* < 0.0001), the right superior occipital gyrus (50-SOG.R) (*P* = 0.004), the right inferior occipital gyrus (54-IOG.R) (*P* = 0.004), and the right paracentral lobule (70-PCL.R) (*P* = 0.023). In MCS patients, the brain regions with increased Nodal clustering coefficients include the left middle frontal gyrus (7-MFG.L) (*P* = 0.006) and the left hippocampus (37-HIP.L) (*P* = 0.006). Brain regions with decreased Nodal clustering coefficient include the left Rolandic operculum (17-ROL.L) (*P* = 0.007), left pericalcarine cortex (43-CAL.L) (*P* = 0.003), right pericalcarine cortex (44-CAL.R) (*P* = 0.009), left cuneus (45-CUN.L) (*P* = 0.008), right cuneus (46-CUN.R) (*P* = 0.003), left lingual gyrus (47-LING.L) (*P* = 0.004), right lingual gyrus (48-LING.R) (*P* < 0.0001), right superior occipital gyrus (50-SOG.R) (*P* < 0.0001), right middle occipital gyrus (52-MOG.R) (*P* = 0.008), left fusiform gyrus (55-FFG.L) (*P* = 0.007), and right fusiform gyrus (56-FFG.R) (*P* = 0.008).

There was no difference in the Nodal clustering coefficient between the VS/UWS group and the MCS group.

**Figure 6:**
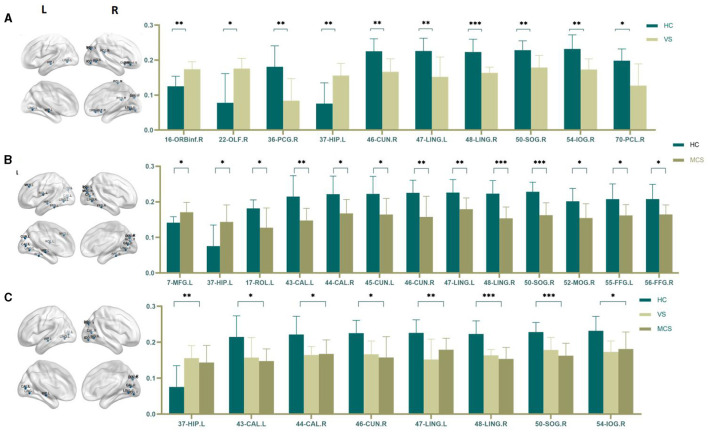
Differences in the Nodal clustering coefficient. **(A)** Comparison between HC and VS; **(B)** Comparison between HC and MCS; **(C)** Comparison between three groups; The 3D brain model on the left presents the localization of brain regions featuring significant differences. The vertical axis indicates the functional connection strength; *P < 0.05; **P < 0.005; ***P < 0.001.

The brain regions with differences among the three groups include the left hippocampus (37-HIP. L) (*P* = 0.003), the left peri talate cortex (43-CAL.L) (*P* = 0.008), and the right peri talate cortex (44-CAL. R) (*P* = 0.006), the right cuneiform lobe (46-CUN.R) (*P* = 0.004), the left lingual gyrus (47-LING.L) (*P* = 0.002), the right lingual gyrus (48-LING.R) (*P* < 0.0001), the right superior occipital gyrus (50-SOG.R) (*P* < 0.0001), and the right inferior occipital gyrus (54-IOG.R) (*P* = 0.007) (as shown in Figure 6, Table 6).

**Table 6:** Statistics of Nodal clustering coefficient.

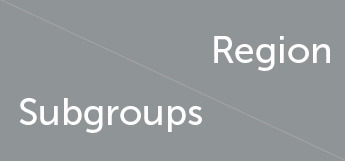	**7-MFG.L**	**16-ORBinf.R**	**17-ROL.L**	**22-OLF.R**	**36-PCG.R**	**37-HIP.L**
HC	0.141 ± 0.017	0.125 ± 0.029	0.182 ± 0.024	0.078 ± 0.084	0.181 ± 0.060	0.076 ± 0.059
VS	-	0.174 ± 0.022	-	0.176 ± 0.029	0.084 ± 0.063	0.156 ± 0.035
MCS	0.171 ± 0.027	-	0.127 ± 0.056	-	-	0.144 ± 0.048
*P*	0.006	0.001	0.007	0.009	0.005	0.003
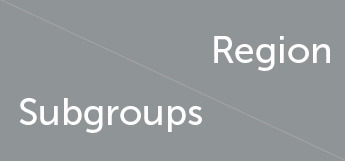	**43-CAL.L**	**44-CAL.R**	**45-CUN.L**	**46-CUN.R**	**47-LING.L**	**48-LING.R**
HC	0.215 ± 0.059	0.222 ± 0.051	0.222 ± 0.049	0.225 ± 0.036	0.226 ± 0.037	0.223 ± 0.036
VS	0.157 ± 0.056	0.165 ± 0.023	-	0.167 ± 0.037	0.152 ± 0.057	0.164 ± 0.016
MCS	0.148 ± 0.034	0.168 ± 0.039	0.164 ± 0.045	0.158 ± 0.058	0.179 ± 0.032	0.154 ± 0.032
*P*	0.003	0.006	0.008	0.003	0.002	< 0.0001
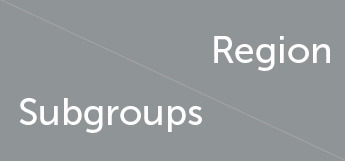	**50-SOG.R**	**52-MOG.R**	**54-IOG.R**	**55-FFG.L**	**56-FFG.R**	**70-PCL.R**
HC	0.228 ± 0.027	0.202 ± 0.036	0.232 ± 0.040	0.208 ± 0.043	0.208 ± 0.041	0.198 ± 0.034
VS	0.179 ± 0.035	-	0.173 ± 0.030	-	-	0.127 ± 0.062
MCS	0.163 ± 0.035	0.155 ± 0.040	0.173 ± 0.030	0.162 ± 0.030	0.165 ± 0.027	-
*P*	< 0.0001	0.008	0.004	0.007	0.008	0.023

Due to different statistical methods for inter-group and intra-group comparisons, the table presents the minimum p value between groups or within groups and the specific values are shown in the text.

##### 3.1.3.4 Nodal efficiency

**Figure 7:**
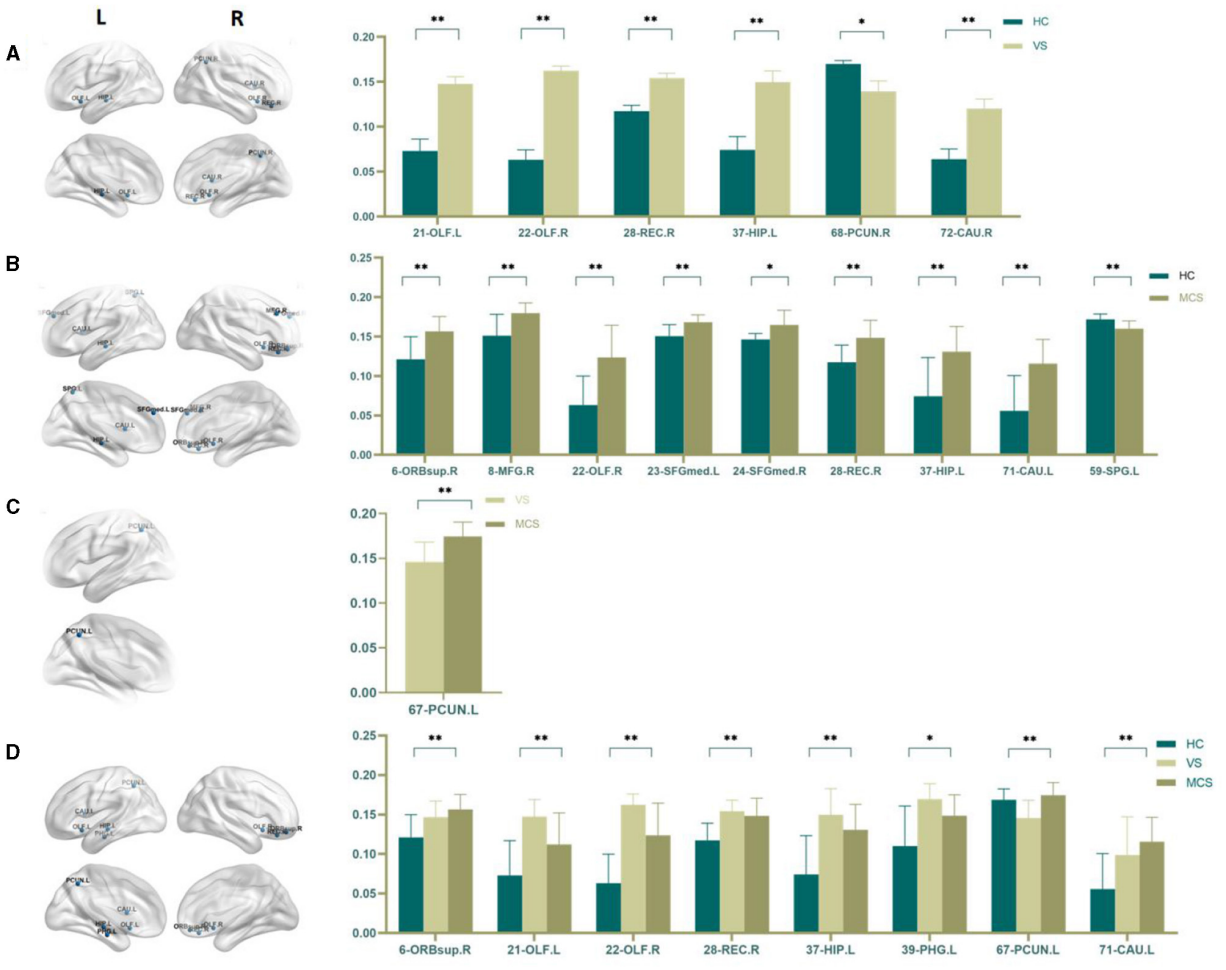
Differences in the Nodal efficiency. **(A)** Comparison between HC and VS; **(B)** Comparison between HC and MCS; **(C)** Comparison between VS and MCS; **(D)** Comparison between three groups; The 3D brain model on the left presents the localization of brain regions featuring significant differences. The vertical axis indicates the functional connection strength; *P < 0.05; **P < 0.005.

Nodal efficiency refers to the communication efficiency between neighboring nodes of a node when it is removed. If a node has high efficiency, it indicates that when it is removed, communication between other nodes in the network will be greatly affected, so this node has high importance in the network. In brain network analysis, node efficiency can be used to evaluate the role and importance of different brain regions in neural information transmission. Compared with the HC group, the brain regions with increased Nodal efficiency in the VS/UWS group included bilateral olfactory cortex (21-OLF.L, 22-OLF.R) (*P* < 0.0001), right rectus muscle (28-REC.R) (*P* = 0.001), left hippocampus (37-HIP.L) (*P* = 0.003), and right caudate nucleus (72-CAU.R) (*P* = 0.004). The brain regions with decreased Nodal efficiency include the right anterior cuneiform lobe (68-PCUN. R) (*P* = 0.042); the brain regions with increased Nodal efficiency in MCS patients include the right orbitofrontal superior gyrus (6-ORBsup. R) (*P* = 0.002), the right middle frontal gyrus (8-MFG. R) (*P* = 0.004), the right olfactory cortex (22-OLF. R) (*P* = 0.001), the left medial superior frontal gyrus (23-SFGMed. L) (*P* = 0.002), the right medial superior frontal gyrus (24-SFGMed. R) (*P* = 0.006), the right rectus muscle (28-REC. R) (*P* = 0.003), the left hippocampus (37-HIP. L) (*P* = 0.003), and the left coccyx. Nucleus striatus (71-CAU. L) (*P* = 0.001); the brain area with decreased Nodal efficiency is the left superior parietal gyrus (59-SPG. L) (*P* = 0.003). The brain area with significant differences in Nodal efficiency between the VS/UWS group and the MCS group was the left anterior cuneiform lobe (67-PCUN. L) (*P* = 0.005), with the MCS group having higher connectivity strength than the VS/UWS group. The brain regions with differences in Nodal efficiency among the three groups were the right orbitofrontal gyrus (6-ORBsup. R) (*P* = 0.004), bilateral olfactory cortex (21-OLF. L) (*P* = 0.001), (22-OLF. R) (*P* < 0.0001), right rectus muscle (28-REC. R) (*P* = 0.001), left hippocampus (37-HIP. L) (*P* = 0.001), left parahippocampal gyrus (39-PHG. L) (*P* = 0.006), left anterior cuneiform lobe (67-PCUN. L) (*P* = 0.004), and left caudate nucleus (71-CAU. L) (*P* = 0.005) (as shown in Figure 7, Table 7).

**Table 7:** Statistics of Nodal efficiency.

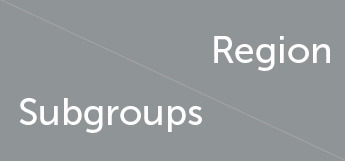	**6-ORBsup.R**	**8-MFG.R**	**21-OLF.L**	**22-OLF.R**	**23-SFGmed.L**	**24-SFGmed.R**	**28-REC.R**
HC	0.121 ± 0.029	0.151 ± 0.027	0.073 ± 0.044	0.063 ± 0.037	0.151 ± 0.015	0.146 ± 0.008	0.117 ± 0.022
VS	0.147 ± 0.020	-	0.148 ± 0.021	0.162 ± 0.014	-	-	0.154 ± 0.014
MCS	0.157 ± 0.019	0.180 ± 0.013	0.112 ± 0.040	0.124 ± 0.041	0.168 ± 0.009	0.165 ± 0.019	0.148 ± 0.022
*P*	0.002	0.004	< 0.0001	< 0.0001	0.002	0.006	0.001
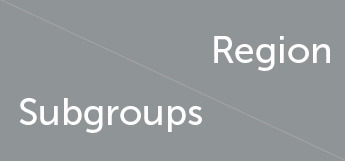	**37-HIP.L**	**39-PHG.L**	**59-SPG.L**	**67-PCUN.L**	**68-PCUN.R**	**71-CAU.L**	**72-CAU.R**
HC	0.074 ± 0.049	0.110 ± 0.051	0.172 ± 0.007	0.169 ± 0.014	0.170 ± 0.013	0.056 ± 0.045	0.064 ± 0.038
VS	0.150 ± 0.033	0.170 ± 0.019	-	0.146 ± 0.022	0.139 ± 0.031	0.099 ± 0.048	0.120 ± 0.028
MCS	0.131 ± 0.032	0.149 ± 0.026	0.160 ± 0.010	0.175 ± 0.016	-	0.116 ± 0.031	-
*P*	0.003	0.006	0.003	0.004	0.042	0.001	0.004

Due to different statistical methods for inter-group and intra-group comparisons, the table presents the minimum p value between groups or within groups and the specific values are shown in the text.

### 3.2 rTMS research

#### 3.2.1 Demographic and clinical characteristics

In this study, 42 DOC patients were included, of whom 2 withdrew from the trial due to changes in their conditions, and finally, 20 patients in each group were included to complete rTMS treatment and included in statistical analysis. Of the 20 participants in the dorsolateral prefrontal cortex (DLPFC) stimulation group, 11 were males and 9 were females. Among them, there were 8 patients with VS/UWS, 11 patients with MCS, and 1 patient with EMCS. Of the 20 participants in the occipital lobe (olfactory cortex-associated brain region) stimulation group, 13 were male and 7 were female. Among them, there were 11 patients with VS/UWS, 8 patients with MCS, and 1 patient with EMCS. There was no significant difference between the two groups in age (F = 0.874, *P* = 0.356), sex (X^2^ = 0.417, *P* = 0.519), or baseline CRS-R score (U=137.50, Z=-1.70, *P* = 0.089) between the two groups (Table 8). There were no adverse reactions or adverse events in this study.

**Table 8:** Demographic and clinical characteristics in rTMS research.

**Subgroup**	**Sex (M/F)**	**Age**	**CRS-R score**
DLPFC	11/9	61.75 ± 14.581	8.50 ± 4.12
Occipital lobe	13/7	59.00 ± 11.088	6.05 ± 4.62
	*X^2^* = 0.417	*F* = 0.874	*Z* = −1.70
	*P* = 0.519	*P* = 0.356	*P* = 0.089

#### 3.2.2 Treatment effect

##### 3.2.2.1 CRS-R grade

**Figure 8:**
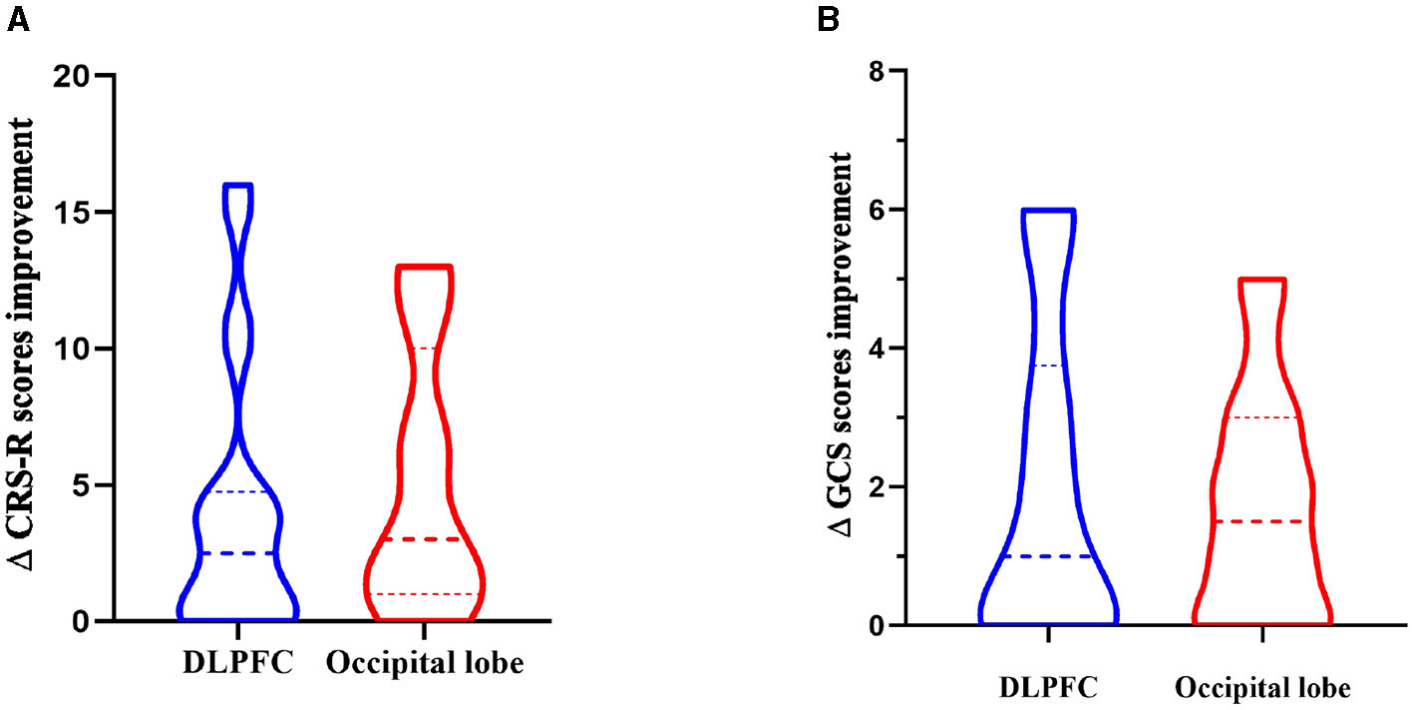
Comparison of changes between the two groups. **(A)** CRS-R scores. **(B)** GCS scores.

Compared with the CRS-R score before treatment, among the 20 patients in the DLPFC stimulation group, the CRS-R score of 14 patients increased, 6 patients had no change, no patient decreased, and the improvement rate of consciousness impairment was 70%. Among the 20 patients in the occipital lobe (olfactory cortex-related area) stimulation group, 17 patients had an increase in CRS-R score, 3 patients had no change, no patient decreased, and the improvement rate of consciousness impairment was 85%. The results showed that the CRS-R score data did not conform to the normal distribution, and the Wilcoxon test was used to analyze the CRS-R scores of the subjects in the two groups before and after rTMS treatment. The Wilcoxon test showed that the CRS-R scores of the two groups were significantly improved after treatment compared with those before treatment (Z = −4.867, *P* < 0.0001). The Mann-Whitney U test was used to compare the difference between the two groups of CRS-R scores, and there was no significant difference in the degree of improvement of CRS-R scores between the two groups (U = 169.5, Z = −0.832, *P* = 0.405>0.05), indicating that the level of awareness enhancement was comparable between the two groups (as shown in Tables 9, 10, Figure 8).

**Table 9:** CRS-R and GCS scores before and after treatment in different groups.

**DLPFC**								**Occipital lobe (olfactory cortex-associated brain region)**							
**ID**	**Sex**	**Age**	**CRSR**		**GCS**		**Diagnosis**	**ID**	**Sex**	**Age**	**CRSR**		**GCS**		**Diagnosis**
			**Score Pre-treatment**	**Score After-treatment**	**Score Pre-treatment**	**Score After-treatment**					**Score Pre-treatment**	**Score After-treatment**	**Score Pre-treatment**	**Score After-treatment**	
1	F	66	9	19	9	14	MCS	1	M	61	17	17	10	10	EMCS
2	F	63	6	6	9	9	VS	2	F	67	14	17	10	10	MCS
3	M	72	6	8	8	8	VS	3	F	68	13	16	9	10	MCS
4	M	65	7	7	7	7	VS	4	M	61	7	14	5	10	MCS
5	M	14	5	9	6	9	MCS	5	F	61	6	19	9	10	MCS
6	M	72	17	17	10	10	MCS	6	F	63	12	12	8	8	MCS
7	F	70	9	10	7	8	MCS	7	F	67	7	12	7	9	MCS
8	F	80	11	16	10	10	MCS	8	M	61	9	11	8	8	MCS
9	F	76	0	0	3	3	VS	9	F	54	7	14	8	9	MCS
10	F	80	10	10	8	10	MCS	10	M	75	6	19	6	9	VS
11	F	63	6	9	7	8	VS	11	F	57	4	15	5	9	VS
12	M	66	7	23	9	15	MCS	12	M	50	4	5	7	8	VS
13	M	66	5	7	5	9	VS	13	M	75	6	19	6	9	VS
14	M	55	16	17	8	10	MCS	14	M	46	3	4	5	5	VS
15	M	49	8	23	8	14	MCS	15	M	59	2	2	6	6	VS
16	M	52	14	18	11	11	EMCS	16	M	52	4	6	5	8	VS
17	F	57	13	24	9	15	MCS	17	M	57	4	15	5	10	VS
18	M	55	5	9	8	11	VS	18	M	58	3	5	3	5	VS
19	M	65	9	9	8	8	MCS	19	M	24	1	2	3	4	VS
20	F	49	7	11	9	10	VS	20	M	64	6	12	8	10	VS
			**IR:70%**		**IR:60%**						**IR:85%**		**IR:70%**		

DLPFC, Dorsolateral prefrontal cortex; F, Female; M, Male; EMCS, Emerging from minimally conscious state; MCS, Minimally conscious state; VS, Vegetative state; IR, improvement rate. The highlighted part of the figure is the number of patients with an improved score.

**Table 10:** Statistical comparison between the two treatment groups.

		**DLPFC**	**Occipital lobe**	***Z***	***P***
CRS-R	Baseline	8.50 ± 4.12	6.05 ± 4.62	−4.867^a^	< 0.0001
	End	12.60 ± 6.57	9.30 ± 6.20		
	Change	4.10 ± 4.99	3.25 ± 4.17	−0.832^b^	0.405
GCS	Baseline	7.95 ± 1.82	6.55 ± 2.19	−4.479^a^	< 0.0001
	End	9.95 ± 2.91	7.75 ± 2.43		
	Change	2.00 ± 2.27	1.20 ± 1.76	−0.014^b^	0.989

^a^Comparison of scores before and after treatment; ^b^Comparison of changes between the two groups.

##### 3.2.2.2 GCS grade

Compared with the GCS score before treatment, among the 20 patients in the left DLPFC stimulation group, the GCS score of 12 patients increased, 8 patients had no change, no patient decreased, and the improvement rate of consciousness impairment was 60%. Among the 20 patients in the occipital lobe (olfactory cortex-related area) stimulation group, 14 patients had an increase in GCS scores, 6 patients had no change, no patients had a decrease, and the improvement rate of consciousness impairment was 70%. The results showed that the GCS score data did not conform to the normal distribution, and the Wilcoxon test was used to analyze the GCS scores of the subjects in the two groups before and after rTMS treatment. The Wilcoxon test showed that the GCS scores of the two groups were significantly improved after treatment compared with those before treatment (Z = −4.479, *P* < 0.0001). The Mann-Whitney U test was used to compare the difference between the two groups of GCS scores, and there was no significant difference in the degree of improvement of GCS scores between the two groups (U = 199.5, Z=-0.014, *P* = 0.989 > 0.05), indicating that the level of awareness enhancement of different stimuli was comparable between the two groups (as shown in Tables 9, 10, Figure 8).

## 4 Discussion

### 4.1 Brain network research

Recovery of consciousness, including the ascending arousal, mesocircuit, and frontoparietal networks, is conceptualized as being contingent upon the re-emergence of dynamic interactions between multiple subcortical and cortical networks.

The recovery of consciousness depends on the functional re-emergence of the brainstem's ascending arousal network (Snider et al., 2019), also known as the ascending reticular activating system (Moruzzi and Magoun, 1949; Steriade, 1996). This must provide sufficient input to the anterior forebrain mesocircuit and frontoparietal networks to depolarize neocortical neurons and facilitate firing.

The anterior forebrain mesocircuit and frontoparietal networks are interconnected, and metabolic activity and functional connectivity within these networks increase as patients' transition from coma to VS/UWS, MCS, a confused state, and eventually to full cognitive recovery (Fridman et al., 2014; Thibaut et al., 2015; Lant et al., 2016).

The fronto-parietal network consists of two subnetworks: the DMN and the executive control network (Raichle and Snyder, 2007; Buckner and DiNicola, 2019). It is known that functional connectivity within the DMN decreases in proportion to the degree of impaired consciousness (Vanhaudenhuyse et al., 2010) and increases with spontaneous recovery (Zhang et al., 2018). In particular, the strength of functional connectivity between the PCC and the medial prefrontal cortex (mPFC) has been shown to predict clinical outcomes in DOC patients (Silva et al., 2015; Liu et al., 2017). The strength of functional connectivity of the DMN is not only crucial for detecting the level of consciousness but is also involved in the process of recovery of consciousness in DOC patients (Coulborn et al., 2021). Some studies have found that the functional brain connectivity of PCC/PCu is directly related to the degree of consciousness impairment, consciousness level, and recovery outcomes in DOC patients, providing neural evidence for the relationship between the functional connectivity direction of PCu and the maintenance of consciousness, indicating that PCu is the central node associated with the consciousness recovery (Guo et al., 2023). In another study, it was found that the fractional amplitude of low-frequency fluctuation in the PCC was reduced in DOC patients and that residual cognitive function was significantly correlated with residual local neuronal activity in the PCC (Salvato et al., 2020).

The research findings of Yu et al. (2021) indicate that DOC can be revealed by abnormalities in directional connectivity patterns at multiple topological scales throughout the brain, and disuptions in directional connectivity patterns may serve as clinical biomarkers for the assessment of functional impairment in patients with consciousness disorders. The decreased global efficiency, reduced clustering coefficient, and increased characteristic path length in DOC patients compared to healthy controls suggest that DOC leads to a large-scale reorganization of brain networks (Yang et al., 2023).

**Figure 9:**
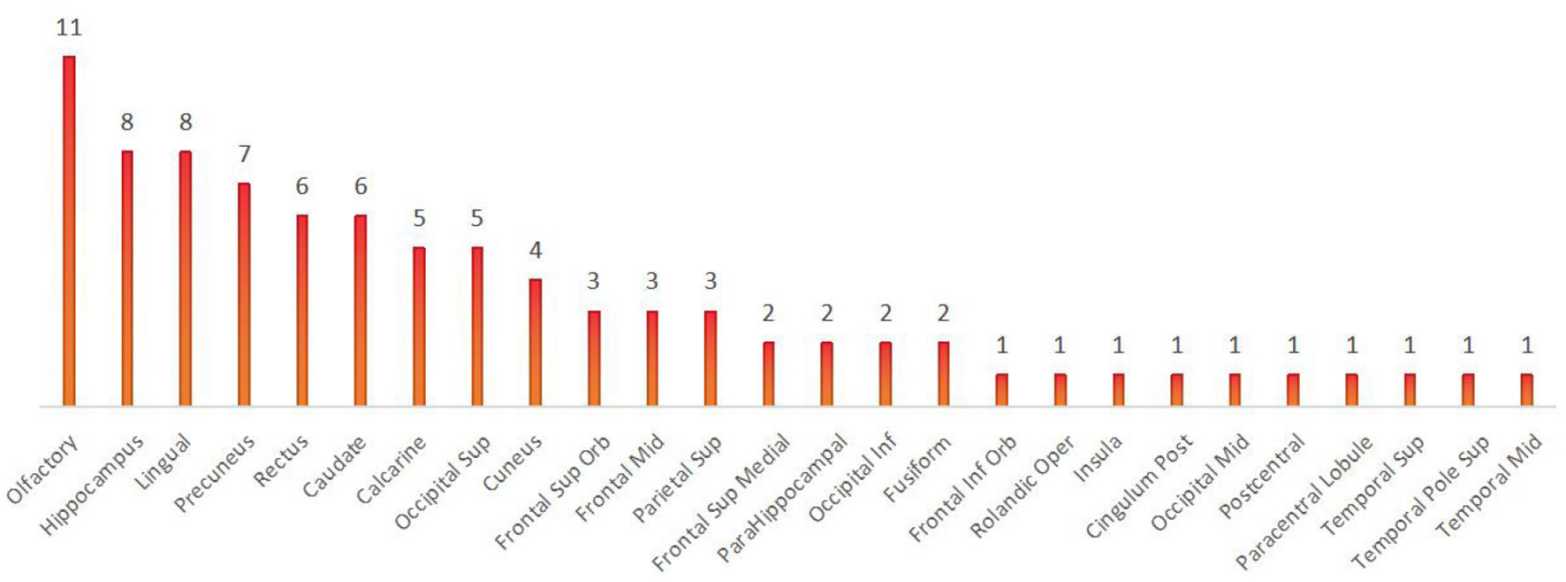
Brain regions with differences among the three groups.

**Figure 10:**
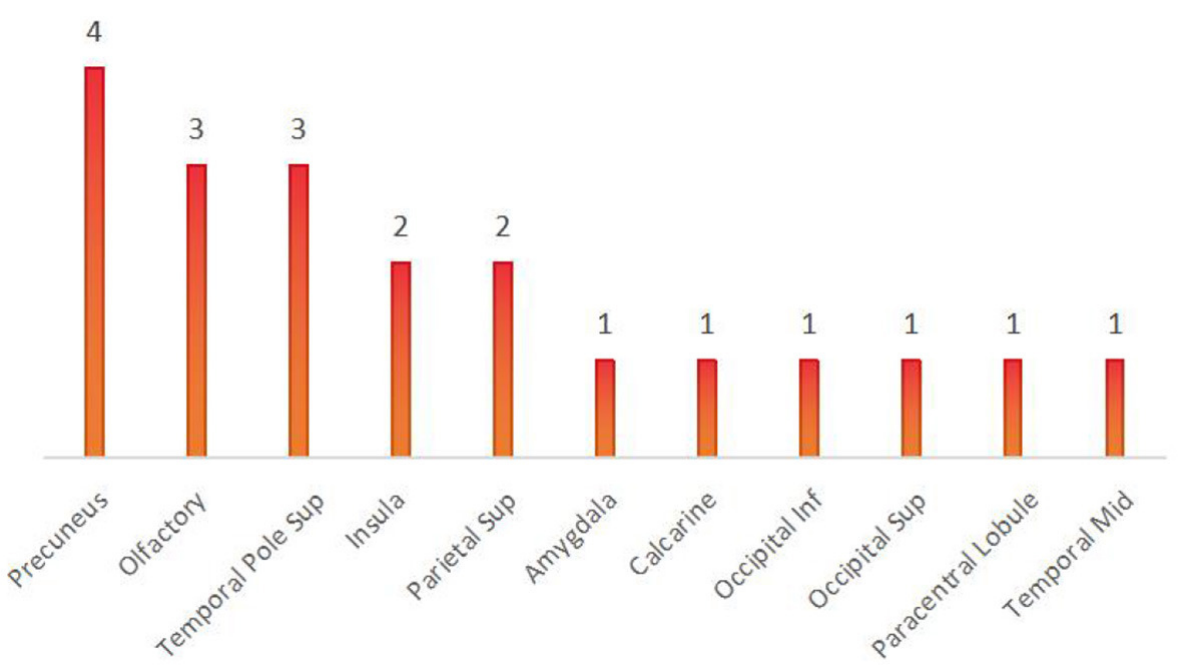
Brain regions with differences between VS and MCS.

In contrast to previous studies, all brain regions with differences in the local brain network, as shown in Result 3.1.3, were counted and summed, and the bar chart was plotted in descending order. We found that the olfactory cortex was the brain region with the highest frequency of differences between the three groups. The precuneus and olfactory cortex are the brain regions with the highest number of differences between the VS and MCS groups. Intuitively, this method can help us understand the changes in brain regions between the different levels of consciousness, providing more objective and effective diagnostic evidence beyond clinical behavioral assessment (as shown in Figures 9, 10).

**Figure 11:**
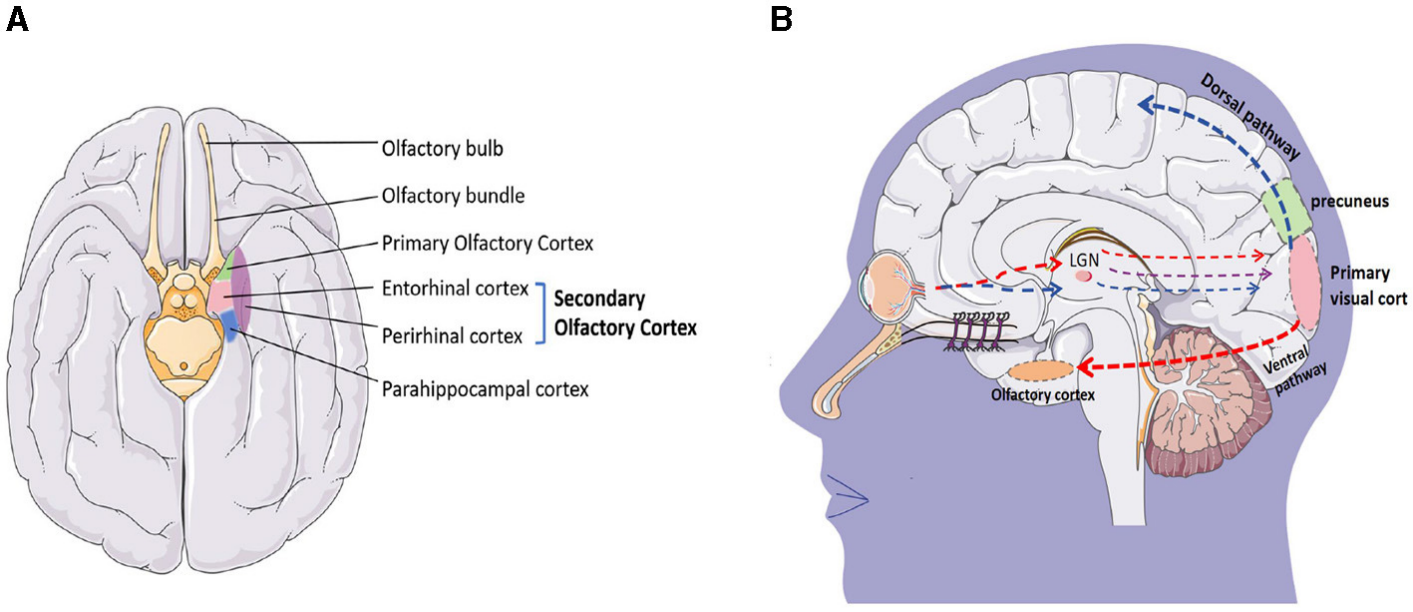
**(A)** Olfactory cortex constitutes; **(B)** visual conduction pathway.

The olfactory cortex is located on the inner side of the temporal lobe, adjacent to the parahippocampal gyrus at the posterior end, and closely attached to the hippocampus on the dorsal side. It is a complex network structure in the brain that is involved in processing and understanding olfaction. It consists of four main regions: the olfactory bulb, pear-shaped cortex, entorhinal cortex, and orbitofrontal cortex (Figure 11A). These structures work together to process olfactory information, including recognizing and distinguishing different odors, linking odors with memory and emotions, and integrating olfactory information with other sensory and cognitive processes.

The production of smell is a complex process. The olfactory epithelium in the nasal cavity contains over 400 olfactory receptors that recognize odors. When odors spread to the olfactory mucosa, odor molecules bind to the olfactory receptors, activating the cAMP pathway. Electrical signals are transmitted along the olfactory neurons to the olfactory bulb, which is the first processing center for olfactory information. After processing by the olfactory bulb, they are projected onto the olfactory cortex. The olfactory cortex is divided into the primary olfactory cortex and the secondary olfactory cortex. The primary olfactory cortex is composed of the anterior pyriform cortex and the surrounding area of the amygdala, which are involved in processing and analyzing olfactory information. They play a crucial role in odor recognition and discrimination. The secondary olfactory cortex is mainly composed of the inner olfactory cortex, located on the inner side of the temporal lobe. It receives input from the piriform cortex and plays an important role in the integration of odor and memory. In addition, the orbitofrontal cortex is a part of the prefrontal cortex that participates in processing and integrating sensory information from olfactory signals. It links odor with emotion and plays a role in decision-making related to olfactory stimuli (Chen et al., 2022).

The olfactory cortex is also a component of the brain's limbic system and is closely related to other structures of the limbic system, such as the amygdala, hippocampus, and hypothalamus. The amygdala participates in the formation of emotional responses (especially fear responses) and memory; the hippocampus forms and stores memory; and the hypothalamus regulates emotional responses. The olfactory cortex is directly connected to the limbic system, and olfactory impulses are transmitted to the cerebral cortex, activating the limbic system and triggering emotional responses. For example, the fragrance of flowers and some perfume in the park will remind us of some memories, people, or things forgotten. At the same time, odors can activate structures such as the hypothalamus, amygdala, and hippocampus to varying degrees, stimulating the brain to release neurotransmitters and helping to alleviate negative emotions such as stress, anxiety, and depression (Mori and Sakano, 2021).

Similar to our research findings, Di Perri et al. reported abnormally high connectivity in the marginal lobe region in VS/UWS and MCS patients. They believe that this high connectivity may be the result of sustained involvement of residual neural activity in the self-reinforcing neural circuit, which can disrupt normal neural connectivity patterns and affect patient consciousness (Di Perri et al., 2013). The results of Yu et al. (2021) further suggest that these abnormal marginal lobe functional connections have prognostic value. Wang et al. used regional homogeneity (ReHo) analysis and found that MCS and VS/UWS patients showed an increase in ReHo in the marginal structure. The total score of the Coma Recovery Revised Scale (CRS-R) in VS/UWS patients was positively correlated with a decrease in ReHo in the left anterior cuneiform lobe, but no linear trend was found in MCS patients (Wang et al., 2022).

Meanwhile, our study also found that the precuneus is one of the important brain regions involved in consciousness change, which is similar to most research results (Cavanna, 2007). Previously, it was widely believed that the precuneus, like the cuneiform lobe, is a visual processing area, but in fact, it involves a wide range of brain functions. In the process of human evolution, the volume and complexity of the precuneus continue to increase, developing higher cognitive functions, including 1. self-awareness (Lyu et al., 2023), 2. memory and situational retrieval, 3. visual-spatial processing, 4. DMNs (Jitsuishi and Yamaguchi, 2021), and 5. multimodal integration (Messina et al., 2023).

The precuneus is widely known for its involvement in visual-spatial processing (Dadario and Sughrue, 2023), and the olfactory cortex is also closely related to visual processing. Although this sounds very surprising, in 1987, F. Scalia implanted the pre-retinal optic nerve into the ipsilateral striatal area of adult frogs and found that regenerated optic ganglion cell axons grew along the front of the olfactory tract. The optic axons form the terminal nerve plexus in the olfactory cortex, lateral geniculate complex, anterior process, parietal, and basal visual nuclei. These observations indicate that regenerated optic ganglion cell axons have an affinity for neurons in the olfactory cortex and neurons in the postsynaptic visual pathway (Scalia, 1987). In addition, studies have found that retinal projections always terminate in the olfactory cortex. When the area is intact or the lesion is cured, retinal projections always terminate in that area but do not enter the amygdala. These findings support the hypothesis that retinal fibers have a specific affinity for the primary olfactory cortex, and the olfactory cortex is also involved in visual information processing (Scalia, 1992).

Visual conduction is very complex, and the primary visual cortex is divided into two pathways: the ventral pathway and the dorsal pathway to process different visual information (Figure 11B). The ventral pathway mainly runs along the inferior temporal lobe (the area where the olfactory cortex is located), and cortical neurons in the inferior temporal lobe have specific responses to complex visual information such as faces, objects, and scenes. Their receptive field occupies a large part of the field of view and does not cover the entire field of view. Professor Guo et al. explored the visual and olfactory dual modes of fruit flies and found that under certain time and space conditions, fruit flies have the function of learning and memory synergy and mutual transmission between different visual and olfactory modes (Guo and Guo, 2005). Afterward, Zeynep Okray and Scott Waddell's team further found that combining color and odor can enhance memory ability in fruit flies, and multisensory learning can enhance the mushroom body Kenyon cells required for both visual and olfactory memory (Okray et al., 2023). Esposti et al. found in their experiments in zebrafish that neurons are projecting into the retina in the olfactory bulb and demonstrated through *in vivo* calcium imaging and pharmacological experiments that olfactory stimulation can regulate the presynaptic calcium channel current of OFF-type bipolar cells through the dopaminergic loop in the retina, thereby regulating their synaptic transmission gain. The integration of olfactory and visual information can have some impact on the decision-making of zebrafish's predatory behavior in bright and dark environments (Esposti et al., 2013). These studies all indicate a close relationship between vision and smell, but further research is needed to improve the relationship between the precuneus and the olfactory cortex, as well as how they affect changes in consciousness.

From an anatomical perspective, the precuneus is located on the inner surface of the parietal cortex between the central para-lobular sensory-motor cortex and the parietal occipital cortex. There is a unique fibrous connection between the precuneus and the temporal lobe, especially between the posterior-anterior cuneiform lobe and the medial temporal lobe (the area where the olfactory cortex is located). The precuneus is considered the core of the DMN due to its involvement in various cognitive processes, and the medial temporal lobe is also a subsystem of DMN, which reflects the consistency of functional brain networks, but further research is needed to improve (Tanglay et al., 2022). Most studies have shown that deficits in consciousness are often accompanied by weakened connections in DMNs (Cavanna and Trimble, 2006), which is consistent with the results of our analysis of local brain network indicators. Overall, the precuneus is a highly interconnected and functionally diverse region of the brain, involved in a wide range of processes related to self-awareness, memory, and perception.

Therefore, this study innovatively found that the olfactory cortex may be an important brain imaging biomarker for distinguishing patients with different levels of consciousness. fMRI studies have further recognized that some DOC patients with unconscious clinical behavioral manifestations may have residual abilities to process “self,” emotions, and even advanced cognitive functions. Patient family members and medical staff can try to stimulate DOC patients from the perspective of smell, taking into account their personality traits and lifestyle habits, to elicit emotional responses as a daily wake-up method. If the patient has an eye-opening reaction, a combination of olfactory and visual stimuli can be used as a means of inducing wakefulness. This study found that the local topological properties of the brain network in DOC patients were damaged to varying degrees, and emerging dynamic analysis methods seem to be more helpful in understanding the essence of consciousness generation, providing better imaging evidence for early identification of consciousness disorders, and further providing a basis for clinical treatment choices for consciousness disorders.

### 4.2 Non-invasive neural regulation—rTMS research

The advent of non-invasive neuromodulation provides a new option for the treatment of DOC, which refers to a treatment method that modifies neural activity by targeting the delivery of electromagnetic stimulation or chemical stimuli to specific parts of the nervous system through specific devices. These include repetitive transcranial magnetic stimulation (rTMS) (Pape et al., 2014), transcranial direct current stimulation (tDCS) (Bai et al., 2017), and others.

TMS uses electromagnetic pulses to induce local nerve depolarization and discharge, and based on the principle of electromagnetic induction, an electric field is formed in the brain to induce depolarized neurons to achieve the effect of regulating cortical excitability. In a double-blind randomized controlled trial (Cincotta et al., 2015), no behavioral improvement was observed in 11 patients with VS/UWS (9–85 months post-injury) after five sessions of rTMS treatment in the primary motor cortex (M1) after 10 min. Another randomized controlled trial (Liu et al., 2016) also reported no behavioral improvement in a group of DOC patients who received repetitive transcranial magnetic stimulation at 20 Hz in the M1 region for approximately 10 min, but there was an increase in hemodynamic function (i.e., cerebral blood flow velocity) in patients in the MCS group and not in patients with VS/UWS. In a single-blind, uncontrolled study (Xia et al., 2017), 20 repeated transcranial magnetic stimulations of the dorsolateral prefrontal cortex at 10 Hz (each lasting 11 min) were performed in 16 patients with DOC and found an increase in CRS-R scores in 5 patients with MCS and 4 out of 11 patients with VS/UWS (36 percent). For rTMS, the prefrontal cortex may be a better target than the primary motor cortex, as all studies on rTMS in the primary motor cortex have shown poor clinical improvement (Pisani et al., 2015).

In 2021, the European Expert Group of the International Union of Clinical Neurophysiology updated the “Guidelines for the Application of Repetitive Transcranial Magnetic Stimulation (rTMS) Therapy,” in which the use of rTMS to stimulate left and/or right DLPFC or M1 high-frequency stimulation in patients with impaired consciousness is recommended, providing a research direction for helping patients with craniocerebral injury to recover consciousness. The guidelines note that although rTMS does not meet the criteria for A/B evidence in other diseases or settings, this does not mean that rTMS does not have significant clinical effects. In this study, some control groups also used DLPFC, which is recommended by the guidelines, to perform high-frequency rTMS stimulation, and the results obtained from brain network analysis—olfactory cortex and precuneus—were compared (due to the deep depth of rTMS stimulation, considering the conclusions of the previous part, we selected the occipital lobe as the stimulation area in this study). Through GCS and CRS-R scores, it was found that the effectiveness of rTMS stimulation therapy for the recovery of consciousness was obvious regardless of the location of the brain region stimulated in this study. The improvement rate of consciousness score in the stimulated occipital lobe group was higher than that in the DLPFC group, but it was not statistically significant, indicating that the effect of rTMS stimulation in the stimulated occipital lobe group was comparable to that in the left-sided DLPFC group. In clinical work, if the patient's forehead is detoxified, the occipital lobe can be used as an alternative site for treatment.

The main function of the occipital lobe is visual processing, which is responsible for receiving, interpreting, and analyzing visual information, allowing us to see and understand the world around us. Impairment of consciousness usually involves a wider range of brain regions, including the prefrontal cortex, thalamus, and brainstem. In this study, the firing coil was placed in the posterior and lower parts of the brain, which may indiscriminately stimulate part of the brainstem region while stimulating the occipital lobe, which plays a crucial role in impaired consciousness because it is a key structure that connects the brain and spinal cord and controls many of the essential physiological functions necessary to sustain life. The brainstem is a relay station for nerve impulses from the spinal cord to the brain, and the intersecting pathways of nerve impulses in the brainstem ensure proper coordination of sensory and motor functions on both sides of the body. Brainstem damage can lead to a loss of sensory and motor function, which in turn affects the state of consciousness. The brainstem plays a central role in maintaining consciousness and wakefulness, and direct stimulation of the occipital lobe of the brain in this study may also have a partial effect on the recovery of consciousness. When scientists monitored the brain activity of healthy people, they found that some specific areas of the cerebral cortex in the parietal, temporal, and occipital lobes at the back of the brain were active, and they are thought to be neuronal structures closely related to consciousness and play an integral role in constituting human consciousness function.

According to the characteristics of the patient's brain network, the brain network is a complex information processing system inside the brain, and each patient has its own unique network structure and functional characteristics. Therefore, by conducting an in-depth study and analysis of the patient's brain network characteristics, we can more accurately understand the pathophysiological state of the patient and then develop a treatment plan that better meets the patient's individual needs. By fine-tuning parameters such as frequency, intensity, and timing of stimulation, we can more effectively activate or inhibit specific brain regions, thereby optimizing the therapeutic effect. This personalized approach to treatment not only improves the targeting and effectiveness of treatment but also reduces unnecessary side effects and risks. At the same time, we need to further use a repetitive transcranial magnetic stimulator with localization function. Through precise positioning, we can avoid stimulation of non-target areas and reduce unnecessary side effects and risks. This provides patients with a safer and more comfortable treatment experience for more precise treatment.

Due to the high cost of magnetic resonance imaging and the high degree of cooperation required by the participants, and secondly, due to the heterogeneous etiology of the patients enrolled in this study, previous studies have demonstrated that different etiologies can lead to different neuropathological changes in patients with DOC (van der Eerden et al., 2014; Lutkenhoff et al., 2015). However, due to the small sample size of this study, we were unable to explore the changes in these graph theory measures in different etiological subgroups, and future studies should expand the sample size for further validation. At present, there are many types of fMRI scan parameters, data analysis, research indicators, etc., and the analysis methods of each research team in the past are different, so it is difficult to conduct meta-analysis. The next step is to develop national and even international multi-center collaborations to develop a unified standard of fMRI scanning and analysis parameters, which will help to overcome these limitations and give us a deeper understanding of consciousness and the neural mechanisms associated with consciousness disorders. On the other hand, this study needs to further combine cerebral metabolic indexes, electroencephalograms, and cerebral blood perfusion to reveal the pathophysiological characteristics of DOC patients from multiple dimensions and perspectives, to understand the condition of DOC patients more comprehensively, to find more valuable information and clues, and to provide new ideas and directions for future research and treatment. At the same time, this study is also further tracking the clinical outcomes of these DOC patients and aims to establish an outcome prediction model for DOC patients based on brain network data and clinical indicators to better predict the outcome of patients and to choose more timely and effective treatment methods for patients. We believe that with the increasing sample size, we will be able to have a more comprehensive understanding of the pathophysiology of DOC patients and more accurately assess the efficacy of rTMS treatment, thereby providing a more reliable basis for clinical diagnosis and treatment.

## 5 Conclusion

The brain network connectivity of DOC patients with different states of consciousness was different, and the most obvious differences occurred in the olfactory cortex and the precuneus lobe. These findings contribute to a better understanding of the functional connectivity and underlying neural mechanisms of brain regions in patients with DOC and provide a promising neuroimaging biomarker for the clinical diagnosis of patients with MCS and VS/UWS. In this study, there were no adverse reactions or adverse events such as epilepsy in the treatment of high-frequency rTMS stimulation of occipital lobe, or DLPFC. High-frequency rTMS stimulation therapy can effectively improve the consciousness level of DOC patients. There was no significant difference in the treatment effect, and both sites had a good effect on restoring consciousness. For patients with frontal cranial valve dissection, occipital lobe stimulation could be selected as an alternative treatment, which provided new evidence for non-invasive neuromodulation. In the future studies, personalized stimulation programs can be used to improve the treatment effect according to the characteristics of the patient's brain network.

## Data Availability

The datasets presented in this study can be found in online repositories. The names of the repository/repositories and accession number(s) can be found in the article/Supplementary material.
