# Supplementary figures and images for: Study of a precise treatment protocol for patients with consciousness disorders based on the brain network analysis of functional magnetic resonance imaging
Source: Front Neurosci. 2024 Sep 16;18:1443478. doi: 10.3389/fnins.2024.1443478 (PMC11439825; doi:10.3389/fnins.2024.1443478)

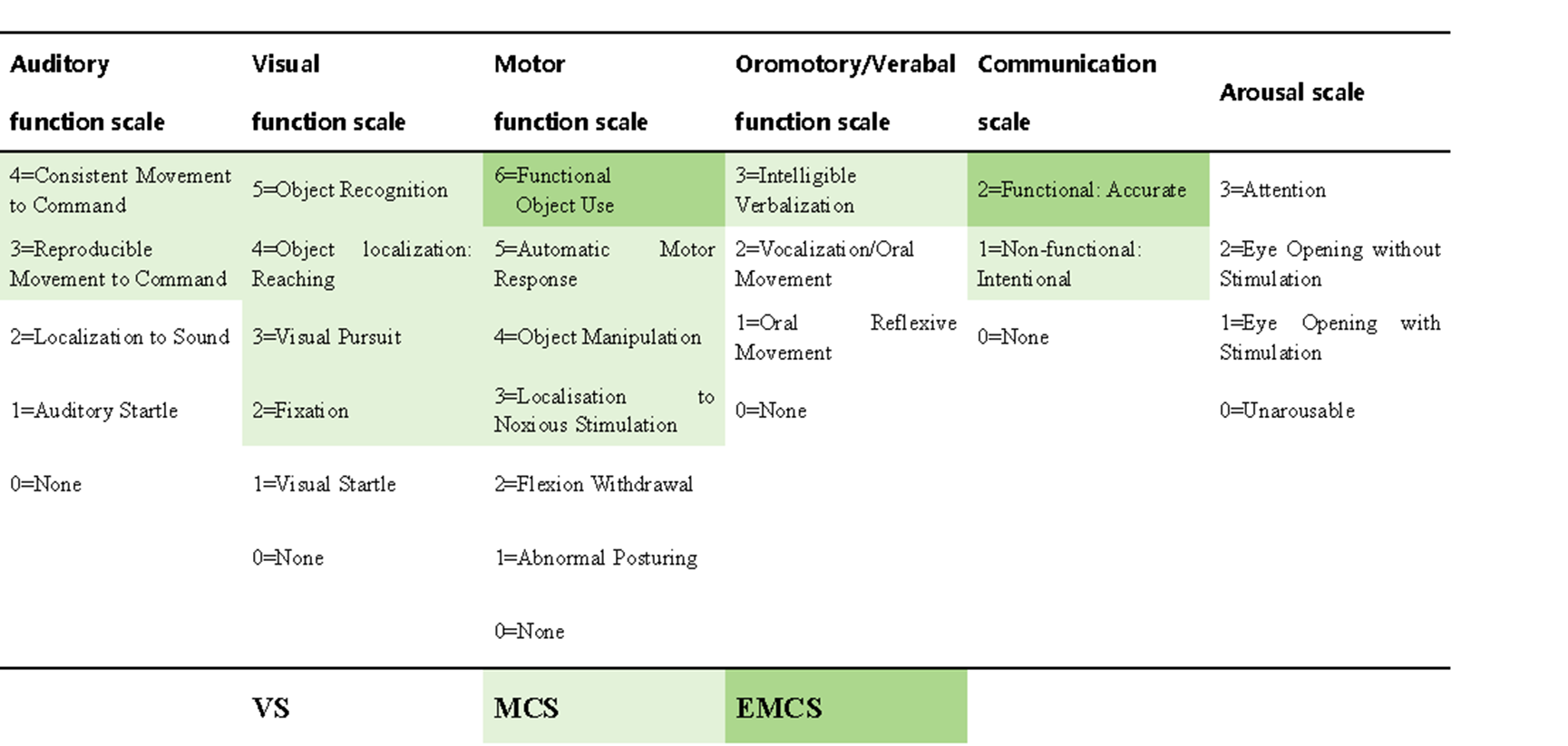

Supplement: Supplementary file 1 [file Image_1.png]

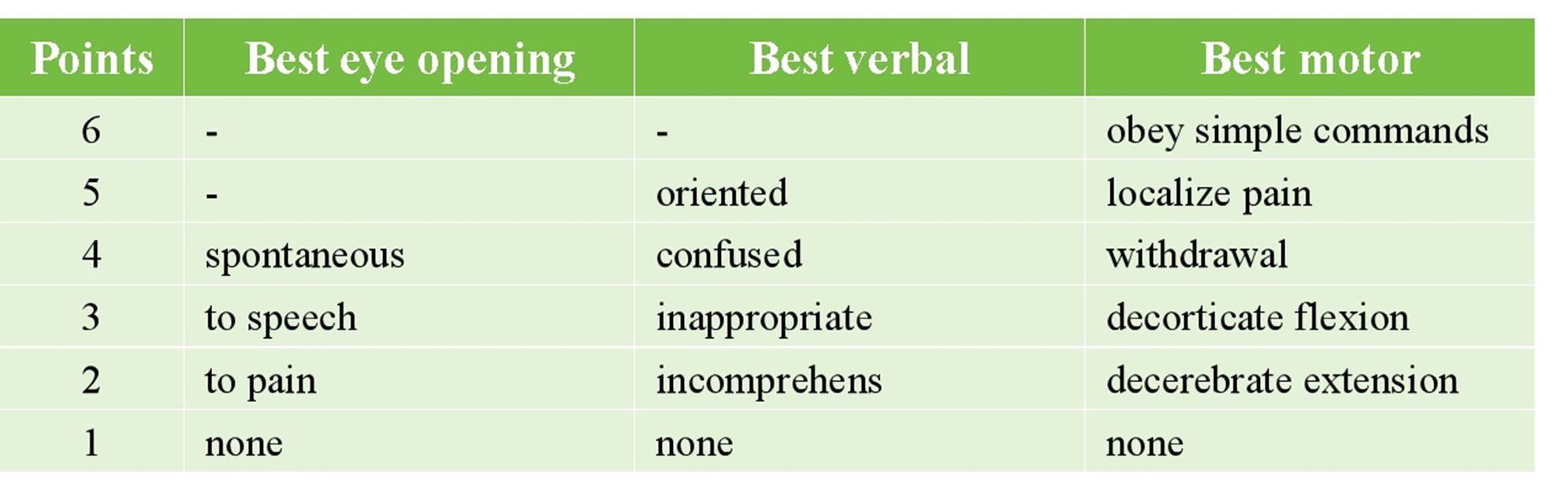

Supplement: Supplementary file 2 [file Image_2.jpeg]

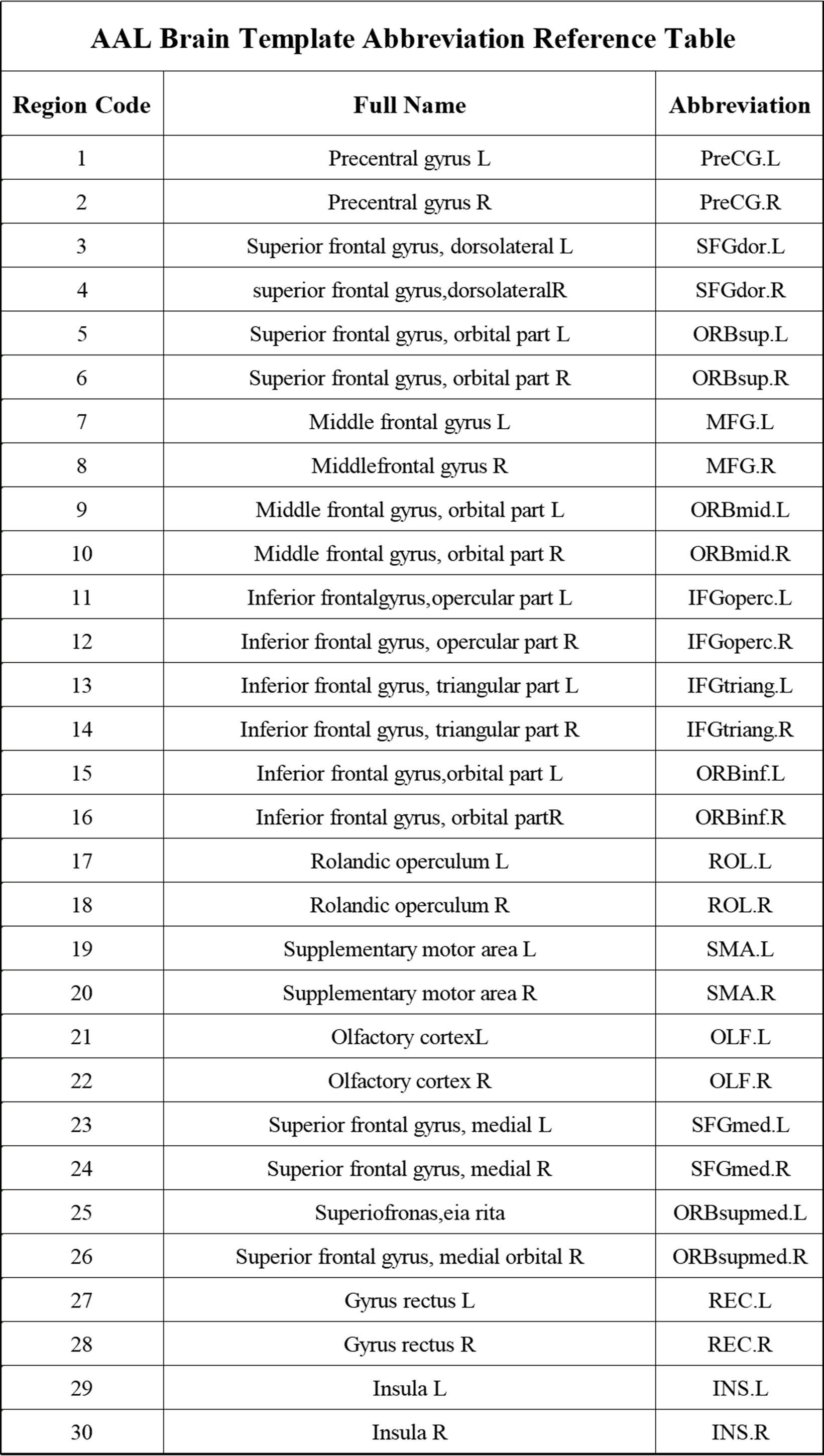

Supplement: Supplementary file 3 [file Image_3.jpeg]

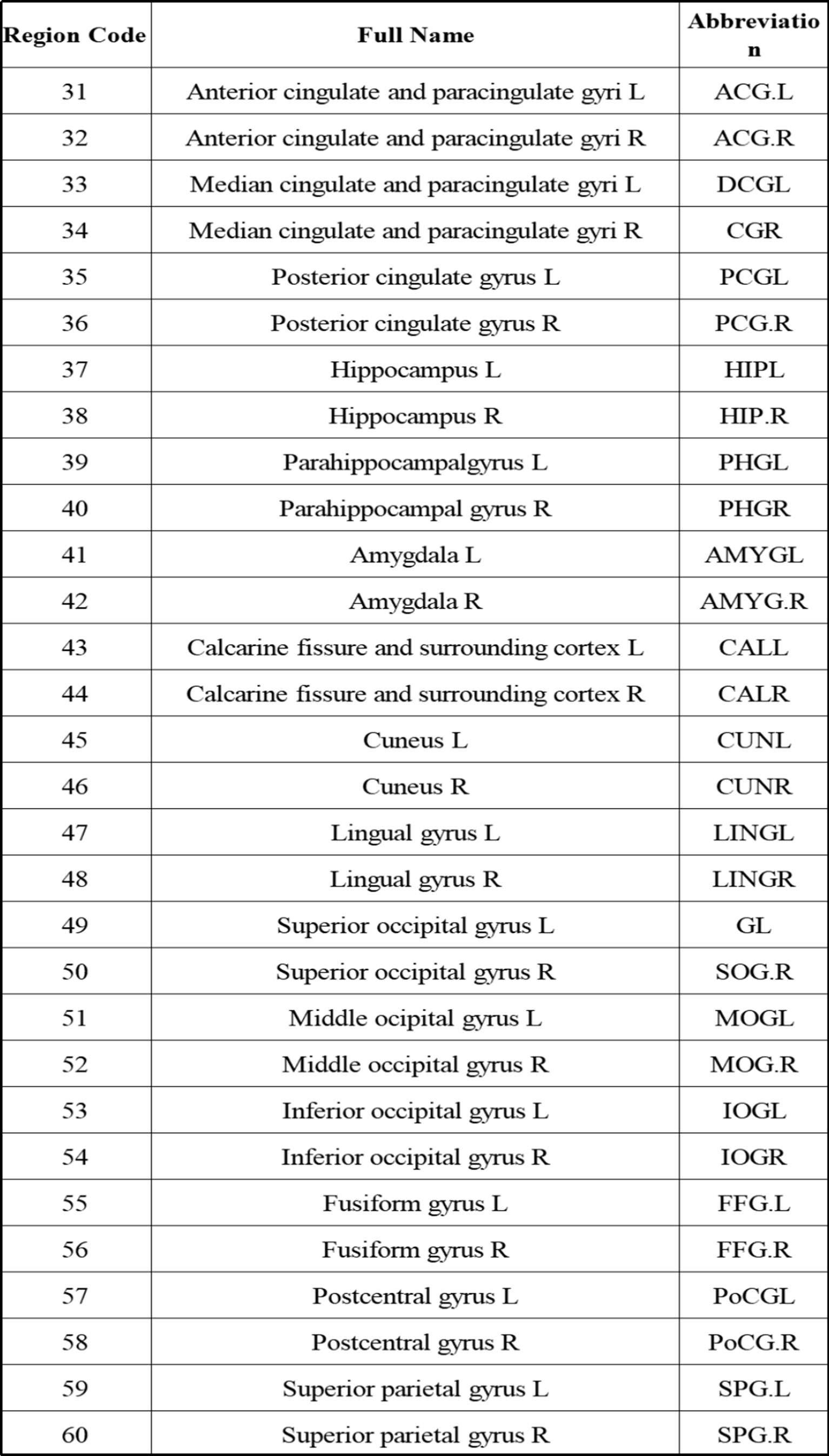

Supplement: Supplementary file 4 [file Image_4.jpeg]

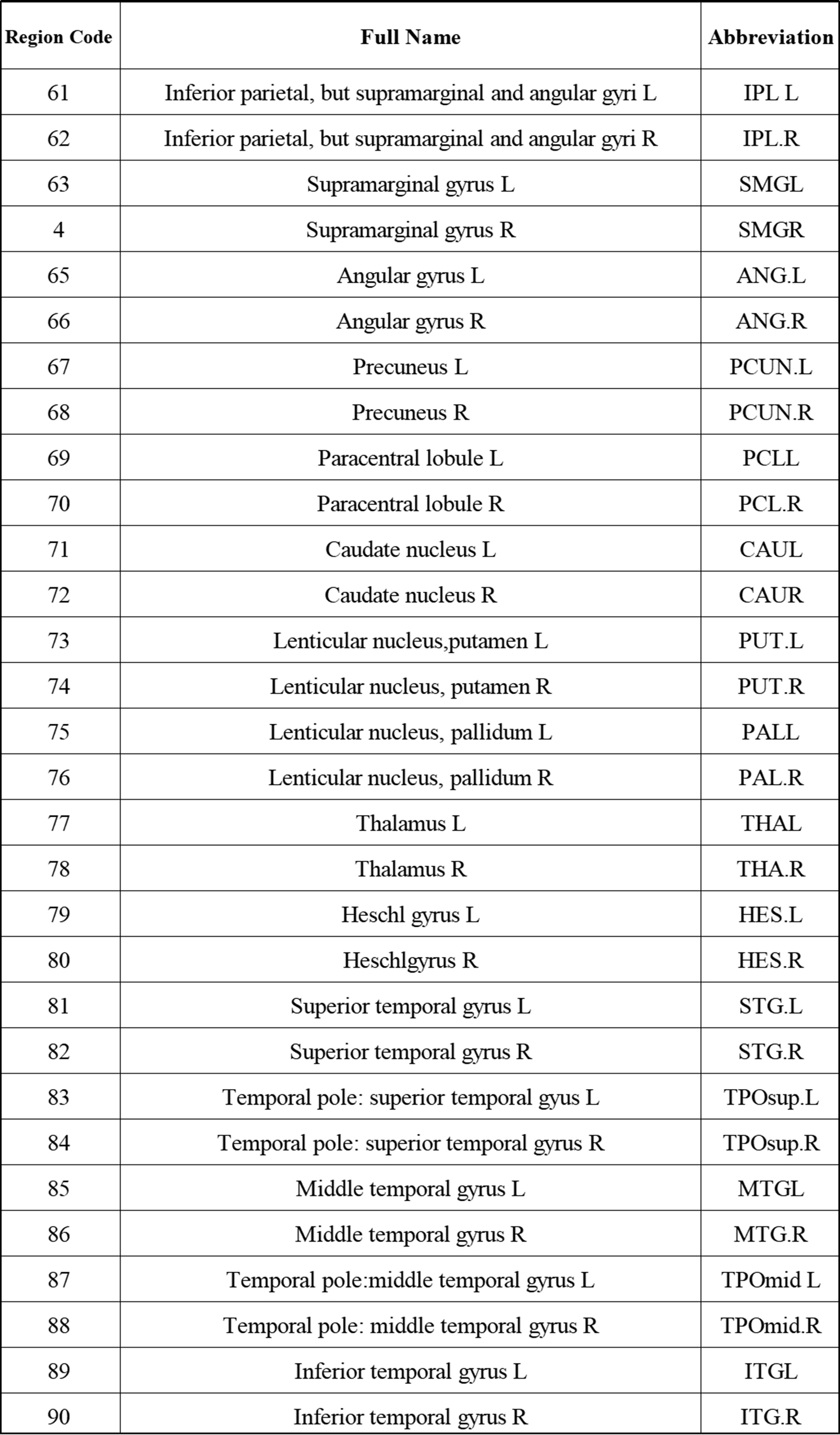

Supplement: Supplementary file 5 [file Image_5.jpeg]
